# Multi-sensor movement analysis for transport safety and health applications

**DOI:** 10.1371/journal.pone.0210090

**Published:** 2019-01-31

**Authors:** Katarzyna Sila-Nowicka, Piyushimita Thakuriah

**Affiliations:** Urban Big Data Centre, School of Political and Social Sciences, University of Glasgow, Glasgow, United Kingdom; Monash University, AUSTRALIA

## Abstract

Recent increases in the use of and applications for wearable technology has opened up many new avenues of research. In this paper, we consider the use of lifelogging and GPS data to extend fine-grained movement analysis for improving applications in health and safety. We first design a framework to solve the problem of indoor and outdoor movement detection from sensor readings associated with images captured by a lifelogging wearable device. Second we propose a set of measures related with hazard on the road network derived from the combination of GPS movement data, road network data and the sensor readings from a wearable device. Third, we identify the relationship between different socio-demographic groups and the patterns of indoor physical activity and sedentary behaviour routines as well as disturbance levels on different road settings.

## Introduction

Recent increases in the use of and applications for wearable technology has opened up many new avenues of research. There are now vast numbers of wearable devices on the market popularly used to record fitness and other leisure activities, monitor personal health and for navigation. Wearable cameras in combination with other sensors allow one’s daily life and environments to be passively and continuously captured in high-resolution image and related data streams and have the potential to generate rich contextual information on people’s movement behaviours.

“Lifelogging”, the term used for a recent phenomenon where people digitally record their daily routines for various reasons and at different levels of detail, has become an active area of research. The definition of lifelogging we use in this paper was suggested by Dodge and Kitchin [1], where lifelogging is referred to as “*a form of pervasive computing*, *consisting of a unified digital record of the totality of an individual’s experiences*, *captured multi-modally through digital sensors*”. Lifelogging may offer the potential to mine or infer knowledge about how we live our lives. There has recently been a convergence of technologies to foster the emergence of lifelogging as a mainstream activity. Computer storage has become significantly cheaper, and advancements in sensing technology allows for the efficient sensing of personal activities, locations and the environment. This is best seen in the growing popularity of the quantified self-movement, in which life activities are tracked using wearable sensors in the hope of better understanding human performance in a variety of tasks [2,3].

Most of the person-level, sensor-based movement analysis known from literature have used various geosensor data: GPS movement data, WiFi data, and the data from Bluetooth and cellular networks [4–6]. Movement analysis can be carried out very accurately, however they do not record essential characteristics of travel behaviour such as travel mode or trip purpose [7]. Furthermore, GPS trackers do not work indoors, and WiFi and Bluetooth beacons do not collect data about individuals but about movement in the area of the beacon, therefore a high percentage of individuals’ daily movement data is missing.

To overcome the problem of enhancing GPS data with contextual information (travel mode or trip purpose), researchers have designed various methods to derive the missing information from GPS trajectories [8–12]. Usually though, most studies using GPS data study “where" and “what" certain people do rather than seek to explain “why" or “how".

In this paper, we consider the use of lifelogging and GPS data to extend fine-grained movement analysis for improving applications in health and safety. A key issue for health monitoring is being able to gather the level of total activity on a daily basis, both indoor and outdoor. From GPS data we are currently able to accurately identify different travel modes and determine activities of individuals when outdoors [11] and define, for example, the levels of active travel (meaning non-motorised modes) per individual [13,14]. As most parts of our daily routines (around 90%) take place indoors, it is also desirable to identify the levels of activity in indoor locations [15]. There is a potential for new transport designs of indoor walking environment in public places such as walking facilities for persons with disabilities [16], designs of public transport accessibility to stations and escalators [17], including capacity analysis of indoor pedestrian facilities [18], evacuation behaviour in buildings [19], crowd movement analysis [20], safety analysis in environments such as parking spaces [21] as well as independence in daily activities [22]. Little is known of the patterns of indoor physical activity and sedentary behaviour routines by various socio-demographic groups. However, awareness of these behaviours is of emerging interest, particularly in respect to public health plans to reduce disease risk factors associated with high levels of sitting time and low levels of physical activity [23].

An area of interest relating to safety applications of these technologies surrounds driver concentration levels for safe driving performance [24–26]. With the growing number of cars on roads, injuries from road traffic incidents are a growing but neglected global crisis [27]. Boredom as well as insufficient levels in task engagement when driving can influence road safety risks, e.g., lack of attention during low traffic periods [28], repetitive daily trips, or semi-automated driving [29]. More and more wearable sensors are being designed to be incorporated into car safety systems to better communicate critical events to drivers without them having to take their eyes off the road [30,31]. Yet, because we can infer where and when in the road network there is disturbance or agitation (using lifelogging data), we can identify locations in the road network that are perceived as potentially hazardous [32,33]. In the context of this paper disturbance/agitation is measured as a difference between the direction a car is heading to and the driver’s body orientation. While identification of hazardous road locations occur from multiple sources of data such accident locations [34,35] and accident severity [36], they do not help to identify the full scope of potential hazards, particularly where avoidance of a hazard may require greater demands of a driver’s skill and reaction time.

The contributions of our research are three-fold:

First, we address the problem of indoor and outdoor movement detection from sensor readings captured by a lifelogger. Second, we propose a set of measures relating to hazards on a road network derived from the combination of GPS movement data, road network data and sensor readings from a wearable device. Third, we examine the relationship between different socio-demographic groups and their indoor and outdoor mobility patterns and raise design implications for hazard avoidance.

The rest of the paper is organized as follows. We first review the recent literature and applications relating to multi-sensor motion detection in Section 2. The data from our multi-modal data collection project and case study are presented in Section 3. In Section 4.1 we propose a method to distinguish indoor and outdoor locations in the data. Next, in Section 4.2, we refine the classification method for the indoor and outdoor movements into different activities and travel modes. In Section 4.3 we propose the methodology to develop an index of the level of driver disturbance or agitation on particular road segments. Different relationships between movement characteristics and socio-demographic groups are studied and presented in Section 4.4. We conclude this work, and present possible future applications and directions of this research in Section 5.

## Related work

This section reviews literature concerning trends in lifelogging and different types of personal wearable sensors, and briefly describes relevant applications and analysis derived from lifelogging data as well as types of road distractions and lifelogging systems to prevent drivers from getting distracted.

A significant portion of the lifelogging research relates to visual lifelogging analysis from image data with a focus on four areas: human interactions, scene understanding, time-based localisation and the activity detection [37]. The number of publications that relate to visual lifelogging has increased exponentially in the last 20 years, which is to be expected given the accessibility of wearable devices. Most wearable cameras on the market such as GoPro, Looxcie and Google Glass have a high temporal resolution whereby an image can be captured between 20 and 60 times per second (these are videos), therefore they can be used to record specific moments and actions. However, the main limitations with using these devices for the type of research described here are battery consumption and storage capacity. There are also other cameras with lower temporal resolution such as the Autographer, Narrative Clip or SenseCam which are more suitable for longer term logging e.g. continuously for a full day, and where knowledge can be inferred of behaviour patterns of the user’s daily life from passive photographs taken of their surroundings. There has been a lot done in the area of object recognition in image processing already, nevertheless there is still room for research around the camera/wearable device user’s environment [37], especially when fusing not only image data but also sensor data. Examples of vision-based and sensor-based activity recognition can be found in wide-ranging studies exploring, for example, health care monitoring advantages, social interactions in stroke survivors and children’s exposure to supermarkets [38–43].

Apart from rich image data, lifelogging devices are often equipped with other sensors allowing the collection of an array of locational and environmental data. Not all lifeloggers have cameras in them either, the most common ones (fitness trackers, pedometers) are simple and equipped with miniature sensors, for example GPS or motion sensors such as accelerometers, gyroscopes and magnetometers which can be worn anytime and anywhere on a wrist or built into smartphones as a multi-sensor-platform [2,44]. Much of the available research focuses on activity recognition using GPS and accelerometer data for the classification of physical activity [45–49], travel mode detection and prediction [50] as well as prediction of accidents and injuries [51].

Recently, wearable fitness trackers have gained a new level of attractiveness due to their ambient data gathering and web-based analysis [52]. These more sophisticated trackers can be equipped with sensors for increasing numbers of personal health-monitoring purposes such heart rate, respiratory rate, blood pressure, blood oxygen saturation, physical activity, proximity to other people, mental attention, fertility, body temperature and muscle activity [53,54]. The wearable devices are easily available as bracelets, wrist bands or clip-ons and many of them can synchronise with a personal computer or smartphone to provide immediate feedback to the user [55]. Automatic behaviour recognition enables a wide variety of applications related to child and elderly care, disease diagnosis and treatment, personal health or sports training, for which motion detection is a crucial component [53].

The increase in range of sources of egocentric mobility data (including sensor readings and images for depicting an activity or an experience) creates major challenges and requires innovative and effective computational solutions [43,56]. One of the most important challenges with the low temporal resolution lifelogging is an automatic identification of highly detailed daily activities [57]. Such information is of high interest to in health-related applications to predict migraines attacks or assure healthy behaviour of patients and individuals of high health risk and monitor elderly people for assistive living [43].

There have been attempts of combining data from external accelerometers such as Actical and images from the wearable device SenseCam to identify a context for certain physical activity [58]. In this era of rising data privacy concerns and regulations, datasets containing personal information as well as unblurred images are not always available for research in full. Lifelogging data when not anonymised might be very disclosive and therefore combining acceleration and strength of magnetic field to obtain orientation as well as other sensor readings associated with images such as brightness, intensity of colours, temperature or motion detection could be potentially used to identify contextual information in human’ behaviour and environmental characteristics of one’s surroundings..

Identifying environmental contexts and certain human behaviours has been of interest recently in developing new-in-car technologies [59–61]. Driving on busy roads is becoming more challenging with higher volumes of traffic. Therefore for greater safety on roads drivers need to become more attentive, correctly perceive their surrounding environment, be able to react with a low response time and make crucial decisions under pressured circumstances [62]. Recently, new in-car driver monitoring systems such as Saab’s Driver Attention Warning System or Toyota’s Driver Monitoring System cope with a number of sources of drivers inattention such as: subjective report measures; driver biological measures; driver physical measures; driving performance measures and hybrid measures [60,63,64]. Most cars however do not have these systems so identifying the potential distractions using other sources of data are of crucial interest. Oviedo-Trespalacios et al. [65] defined types of inattention in three categories: cognitive distraction, visual distraction and manual distraction. Cognitive distraction happens when a driver is not mentally engaged with his/her driving tasks, and not aware of sudden changes in the surrounding environment. A cognitive distraction could occur despite of a correct seating position of a driver. A visual distraction is when a driver is not looking at the road and therefore does not see changing driving conditions. Often these are related to lack of attention due to a secondary task such as texting, looking at mobile phone or a navigation device or just looking around while driving. The third type—manual distraction is when a driver has an incorrect hand position on a steering wheel while driving [66].

Recent research has suggested that driver distraction is a major cause of vehicle collisions [67]. To reduce the safety risk, it is crucial to fundamentally understand the distractions most likely to affect driver road situation awareness [68] and to do this we will aim to develop an index based on multi-sensor data to describe disturbance level on certain road links.

Our primary motivation is to understand ways of combining multisensory data to understand spatiotemporal behaviour and the possible health and safety applications for various socio-demographic groups. To the best of our knowledge there has not been a sensor fusion approach where GPS movement data and sensor data from lifeloggers have been used in combination to identify indoor and outdoor mobility patterns of individuals. Additionally, by defining driver disturbance (agitation) indices we could show a way to monitor potential driving hazards with the objective of informing road network design and operational policies.

## Data and case study

In this study we use the Integrated Multimedia City Data (iMCD) platform that covers the Greater Glasgow urban area, UK. The iMCD data is multi-modal in nature and currently consists of seven strands of data: participant survey with travel diary and activity diary, Internet Information Retrieval on several sources of social media and local and national news websites, remote sensing data, sensor data, specialised private sector datasets as well as background data such as Census data. This type of multi-strand data had been previously noted as being useful in study transport and mobility in the wider urban context [14,69]. The study uses the iMDC participant survey data which consists of three parts: a questionnaire-based survey, personal sensor survey and an activity diary. The survey was designed to provide reliable up-to-date information on Glasgow households. A sample of participants from the main survey took part in a sensor survey which consisted of the collection of GPS movement data along with life-logging data and keeping an activity diary [70]. In this research we use the social survey responses (described in more detail below) and the travel-related datasets: travel diary, activity diary, GPS movement data and lifelogging data from 142 individuals who carried both devices for a satisfactory length of time as well as filled the travel and activity diaries in which can be used as a ground truth. The described data sets had the ethics approved by Ethics Committee for Non-Clinical Research Involving Human Subjects for University of Glasgow (Application Number: 400140085).

### Socio-demographic survey

The iMCD survey is a cross-sectional survey based on a sample of the general population (2095 people from 1509 households) in private residences across the eight local authority areas of Glasgow and Clyde Valley. The survey fieldwork was run by Ipsos MORI and took place between 15 April 2015 and 21 November 2015. The survey was designed to provide trustworthy and current information on Glasgow households, asking about attitudes, beliefs, education, economic status and ethnicity as well as daily routines and and activities. Furthermore, the participants were all asked to record a travel diary over a 24-hour period. The resultant dataset consisted of data from 83 women and 59 men covering a variety of employment statuses (full-time employed, part-time employed, unemployed, self-employed, looking after home, permanently retired, in higher education and unable to work). The sample consisted of 14 people under 21 years old, 100 between 21–65, and 28 older than 65. The average size of household for the participants in the study is 2.57 with 75% owning a driving licence. The average BMI is 26.53 which means that the population taking part in the survey is slightly overweight (According to NHS [2018], BMI up to 24.99 is considered as healthy [71]). Based on the GPS data analysis where we classified the traces into modes and activities we discovered that on average people walk 0.45h a day outdoors.

### GPS data

GPS movement data were collected for seven consecutive days with an interval of 5 seconds. The Transmit 747 ProS GPS tracker was chosen as the most suitable for the project (details about the device can be found in [72]). The device is a standard GPS logger which stores various movement parameters and movement trajectories of objects carrying a device. GPS data were collected by 333 individuals in total providing 6,433,150 GPS data points.

### Lifelogging data

**Fig 1:**
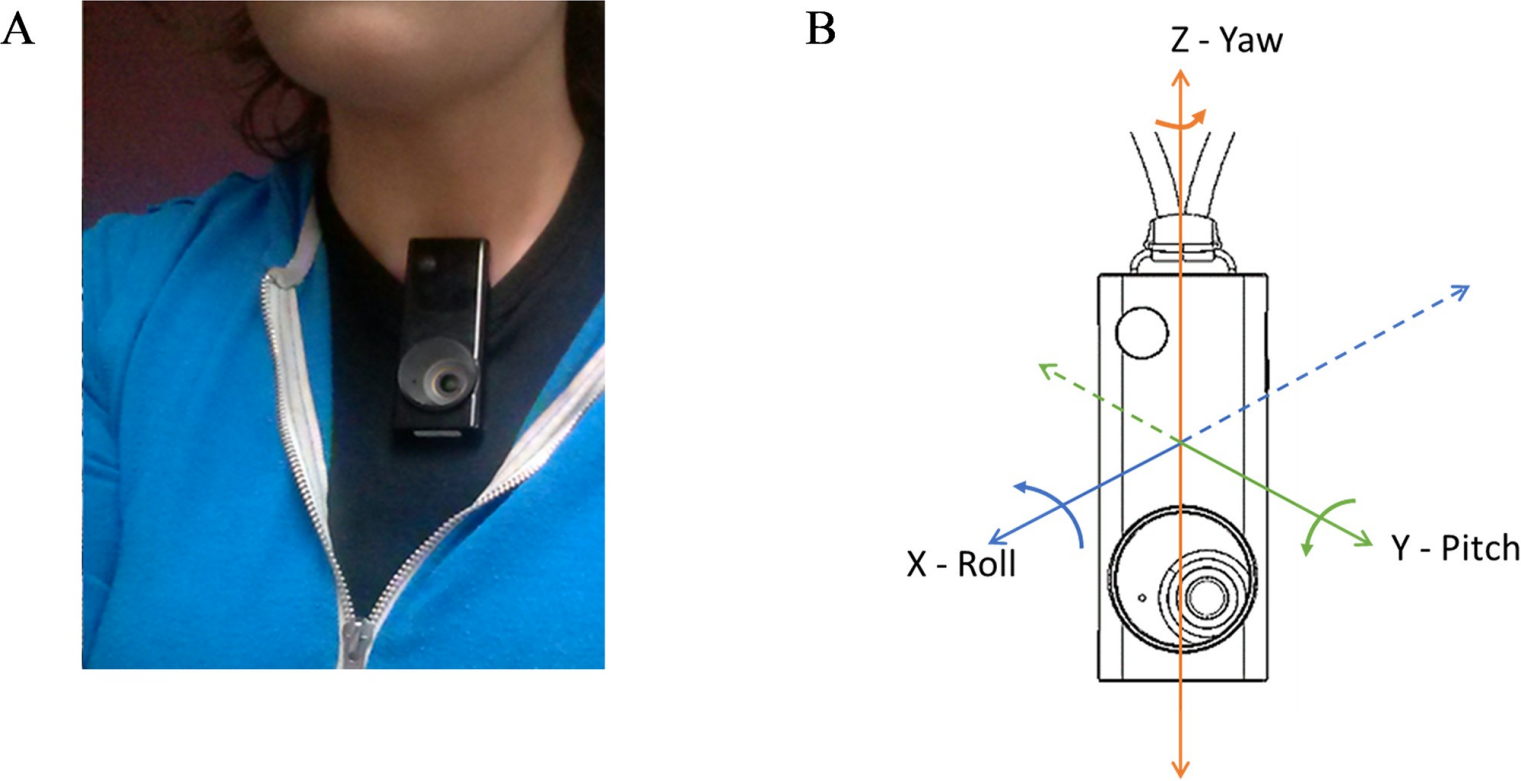
a). Autographer—lifelogging device. b) Orientation determined by Autographer manufacturer using magnetic field and acceleration where Yaw is an Azimuth (0–360 degrees), Roll (-90-90 degrees) and Pitch (-180-180 degrees).

Along with the GPS devices, a sample of participants (223 individuals) carried a lifelogging device (Autographer—see Fig 1a) in order to collect images over two days at time interval of 5 seconds. Participants were instructed to wear these devices clipped to the front of their chest. This strategy also provided the best angle for all types of photo conditions, and also allowed the device to be responsive to body movements identifying the direction person was heading. Every lifelogging image generates a set of associated sensor readings (the sensors are accelerometer, motion detector, magnetometer, thermometer, GPS sensor and a brightness detector as described in Table 1).

**Table 1:** Types of Autographer’s sensors, measurements and measurements’ units.

No	Type of sensor	What is measured	Readings and Units	Comments
1	Accelerometer	Linear Acceleration in X, Y and Z direction including the gravitational force in g (earth and its acceleration equals 9.8 m/s^2^)	Readings for 3 axes: aacx; accy; accz	When device rests on a table then, aacx, aacy are close to 0 and aacz = -1 (gravity pulling down)
2	Magnetometer	Ambient geomagnetic field in uT micro Teslas	Readings for 3 axes: magx; magy; magz	The x,y,z represent the strength and direction of the magnetic field relative to the device
3	Colour Sensor	Light intensity and luminance	RGB readings: red; green; blue, Light sensor reading for luminance indicating brightness: lum,.	
4	Temperature	Measures the ambient temperature	Temperature reading in Celsius degrees Tem	
5	GPS	Location of the Autographer	Latitude and longitude, altitude readings: lat; lon; alt	These are readings which are related to the GPS sensor. For our project we had this setting turned off as it takes more than 20 minutes for a device to fix the position.
6	PIR (Motion Detector)	Detects moving objects using infra-red lights	No known readings	No values
7	Accelerometer and Magnetometer	Orientation determined by Autographer’s manufacturer using magnetic field and acceleration where Yaw is an Azimuth (0–360 degrees), Roll (-90-90 degrees) and Pitch (-180-180 degrees).		

All wearable devices were set on the same computer, which was time synchronised with a world atomic clock, ensuring time synchronisation across all GPS and lifelogging devices [58]. The device was either attached to a clothing on a chest or was hanging on a lanyard around one’s neck. The placement of the device was chosen to afford a wide spectrum of visibility while being comfortable to use throughout the collection period.

## Methodology: Indoor-outdoor activity classification

In this paper we develop a framework for activity recognition both indoor and outdoor by using data associated with image sensors. The sensor data used are: acceleration, magnetic field strength, orientation, luminance and temperature. In this section the objective is to determine from sensor data whether a participant is indoor or outdoor at specific time and the travel mode they were undertaking. To do so, first a training dataset from a full GPS-lifelogging set was created and then manually annotated. The resulting set of data was used to train the models used in this paper. The images themselves were not used in modelling but were used as ground truth data.

### Training data

To create a training set we followed a set of steps, where we first considered all the participants with a parallel continuous coverage of activities recorded by GPS and lifelogging devices. From the 333 people who carried a GPS device, 222 carried a lifelogging device but as some did not have continuous coverage of data from both devices, we used only data from 185 participants. To establish a representative sample of participants for the manual annotation we clustered the whole dataset from the social survey (2095 individuals from which 1509 filled in a travel diary) into 9 clusters (the optimal number of clusters derived from inspecting a dendrogram) using the k-modes clustering method which is an extension of the k-means method [73]. Instead of distances, this method uses a dissimilarity measure to deal with categorical objects and instead of means, it uses modes (a vector of elements that minimises the difference between the vector and other data). In this process we used the following set of variables linked from the social survey and travel diary: age, gender, working status, information whether a person holds a driving licence, number of trips and distance travelled. In the next step nine representative individuals with the highest amount of lifelogging and GPS data were selected from the identified clusters. The identified sample consisted of 19,114 images with corresponding sensor readings for each of the images.

#### Creation of manual annotation

These images had manually annotated travel mode/activity (sitting, standing, walking, running, cycling, lying, driving, being driven and others), social interactions (number of people visible on each image excluding a person carrying the device), information whether an image was taken outdoor or indoor, as well as more specific activity details such as cooking, ironing, reading a book, writing and so on. Data obtained from a lifelogger are characterised with lower temporal resolution of sensor data in comparison to pedometers or physical activity trackers. The average time interval between readings is around 7 seconds whereas for some devices such as pedometers or Fitbits in can be 10 milliseconds. For this reason, for training and modelling purposes the travel modes and activities were reclassified into more general classes: walking indoor, walking outdoor, sitting indoor, sitting outdoor, driving outdoor and other indoor activities combined into one category. It has to be noted that potentially a person in the car could be a passenger of a driving car rather than a driver himself/herself. Among the data from 185 participants (almost 140 000 images) there were only 43 identified images with someone who was in the car and was not a driver of the vehicle at the same time. Furthermore, not having enough data to train the model does not allow us to establish a way to differentiating between a driver and a passenger of a vehicle and we list this as a potential limitation of this study.

#### Description of the training data

**Fig 2:**
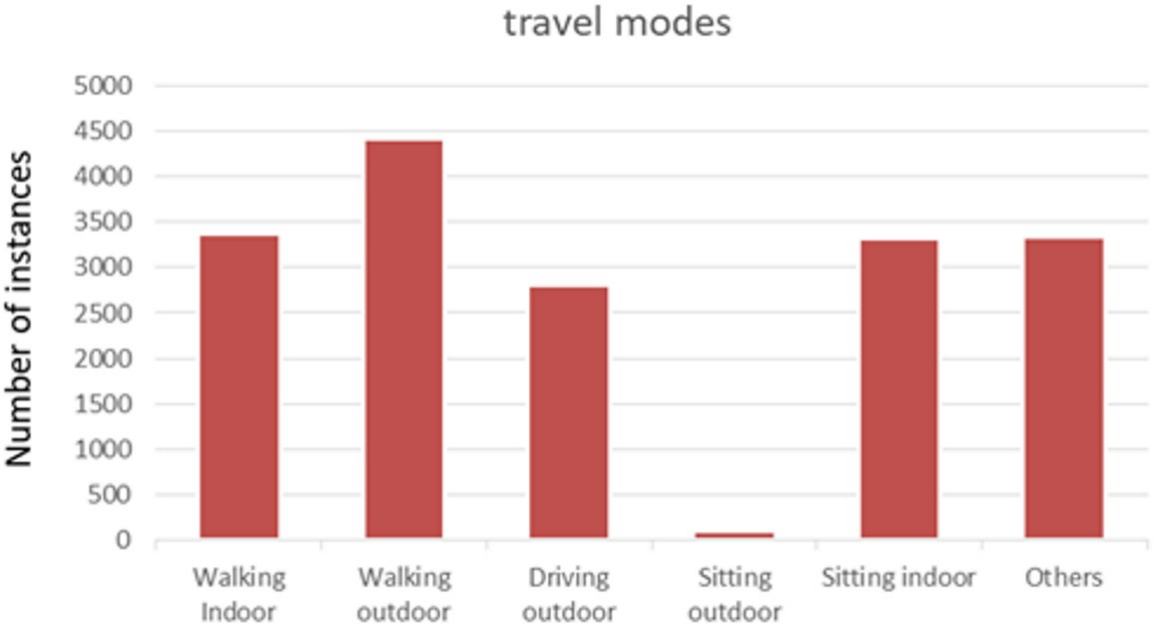
Travel modes in manually annotated set of images.

Table 2 presents an example structure of the dataset consisting of image sensor readings. In Table 3 we present an example set of annotated attributes. Fig 2 presents the distribution of annotated activities within the training set of 19,114 images.

**Table 2:** Data structure for lifelogging dataset. Acceleration and magnetic field strength are calculated from initial sensor readings (Table 1).

Image ID	Timestamp	Accel. (g)	Magnet. (micro Tesla)	Luminance	Temperature (Celsius °)	Orientation (°)
1234	12/09/2015 12:37:08	0.9779	67.5891	395	18.1	185.1700
1235	12/09/2015 12:37:15	0.9877	56.8252	365	18.1	185.1582
1236	12/09/2015 12:37:22	1.3254	22.6354	958	18.2	185.9853
1237	12/09/2015 12:37:29	1.0143	35.2547	945	18.1	180.2584

**Table 3:** Table of assigned labels to each of the images.

Image ID	Timestamp	Location I-O	Activity	Travel mode	Reclassified activity and mode
1234	12/09/2015 12:37:08	Indoor	Walking	Walking	Walking Indoor
1235	12/09/2015 12:37:15	Indoor	Walking	Walking	Walking Indoor
1236	12/09/2015 12:37:22	Outdoor	Walking	Walking	Walking Outdoor
1237	12/09/2015 12:37:29	Outdoor	Walking	Walking	Walking Outdoor

### The random forest

To classify sensor data into outdoor and indoor locations as well as travel modes we used Random Forest, a multiple decision tree classifier that improves on the classification accuracy of a single-tree classifier by combining the bootstrap aggregating (tree-bagging) method and randomization in the selection of the partitioning data nodes of the decision tree [74,75]. The assignment of a new observation vector to a class is based on a majority vote of the different decisions provided by each tree within ‘the forest’. Developments and recent advancements in Random Forest (RF), as well as detailed explanations of how the model works can be found in Fawagreh et al. [76].

#### Calculation procedure

First a set of variables most suitable for indoor and outdoor activity detection was chosen: acceleration, magnetic field strength, orientation as well as time interval, time of the day, total time of recording and temperature. This set of variables was chosen based on Gini importance selection criteria which measures the impurity of data by splits of a given variable. If the variable is useful, it tends to split mixed-label nodes into pure single class nodes [76]. In its general form, it can be calculated as:

$$Gini(t)\;=\;1-\sum_{i\;=\;1}^{N}{P(C_{i}|t)}^{2}$$ (1)

where *t* is a condition, *N* the number of classes in the data set, and *C*_*i*_ is the *i*th class label in the dataset. Next, we trained and tested the model using 17,379 randomly selected (90% of the manually annotated, balanced dataset) sensor records using 200 randomised decision trees with 5 variables tried at each split. To establish the number of trees we ran Random Forest with different tree number values (100, 200, 300…., 1,000) and the recorded Out of Bag error rate (OOB = 0.69%) reached the minimum with 200 trees used. The training set (70% from the initial 90% from the full dataset) contained a known output that the model learned from in order to be applied more generally on other data. The test dataset consisted of the remaining 30% of the initial 90% of the full dataset and was used to test our model’s prediction. Furthermore, to additionally verify the model we predicted the modes for the initial 10% of the full dataset and compared them against the ground truth from manually annotated images. The results of these attempts are presented in Tables 4 and 5.

**Table 4:** Evaluation results for 90% manually annotated data where 70% were used as training and 30% as a testing dataset.

	Walking Indoor	Walking outdoor	Driving outdoor	Sitting outdoor	Sitting indoor	Others	Precision	Recall	F-measure
Walking Indoor	3009	301	3	0	0	59	0.875	0.892	0.884
Walking outdoor	252	4109	45	1	1	3	0.911	0.932	0.921
Driving outdoor	1	17	2721	14	2	76	0.964	0.961	0.963
Sitting outdoor	0	0	8	87	0	12	0.713	0.813	0.760
Sitting indoor	143	65	0	8	3054	56	0.985	0.918	0.950
Others	32	16	45	12	45	3182	0.939	0.955	0.947
Precision	0.875	0.911	0.964	0.713	0.984	0.939			

**Table 5:** Evaluation results for 10% of manually annotated data which were not taken into consideration during the training procedure.

	Walking Indoor	Walking outdoor	Driving outdoor	Sitting outdoor	Sitting indoor	Others	Precision	Recall	F-measure
Walking Indoor	310	21	0	0	0	6	0.865	0.919	0.891
Walking outdoor	32	391	5	0	0	13	0.924	0.887	0.905
Driving outdoor	0	3	268	1	0	8	0.961	0.956	0.959
Sitting outdoor	0	0	0	9	1	1	0.410	0.813	0.545
Sitting indoor	14	7	0	5	301	6	0.976	0.906	0.940
Others	2	2	6	6	6	311	0.904	0.935	0.919
Precision	0.865	0.924	0.961	0.410	0.976	0.904			

#### Evaluation

We evaluated the performance of each classifier using the Precision, Recall, and F-score metrics. Precision (also called positive predictive value) is the fraction of retrieved instances that are relevant, while Recall (also known as Sensitivity) is the fraction of relevant instances that are retrieved.

$$Precision\;=\;\frac{TP}{TP+FP}$$ (2)

$$Recall\;=\;\frac{TP}{TP+FN}$$ (3)

where TP is a true positive, FP is a false positive, FN is a false negative instance.

Furthermore, to show the overall accuracy of our results we use F-measure [77], which can be written as:

$$F-measure\;=\;\frac{2*Precision*Recall}{Precision+Recall}$$ (4)

These metrics provide detailed information about how the algorithm performs on each class.

#### Results

The results show that the RF classifies data with high precision and accuracy (Tables 4 and 5). The sitting indoor and driving activities are characterised with the highest precision and accuracy of classification whereas sitting outdoor has the lowest accuracy and precision due to the lowest number of instances input into the training set. Even though in our study the sensor data are sparse (longer recording interval period—data are being collected every 6 seconds) the study demonstrates similar accuracy rates for activity classification as Reddy et al. [2013] who employed smartphones with much lower data capture interval rather than lifelogging devices.

### Contributions to indoor-outdoor classification and limitations of lifeloggers

**Fig 3:**
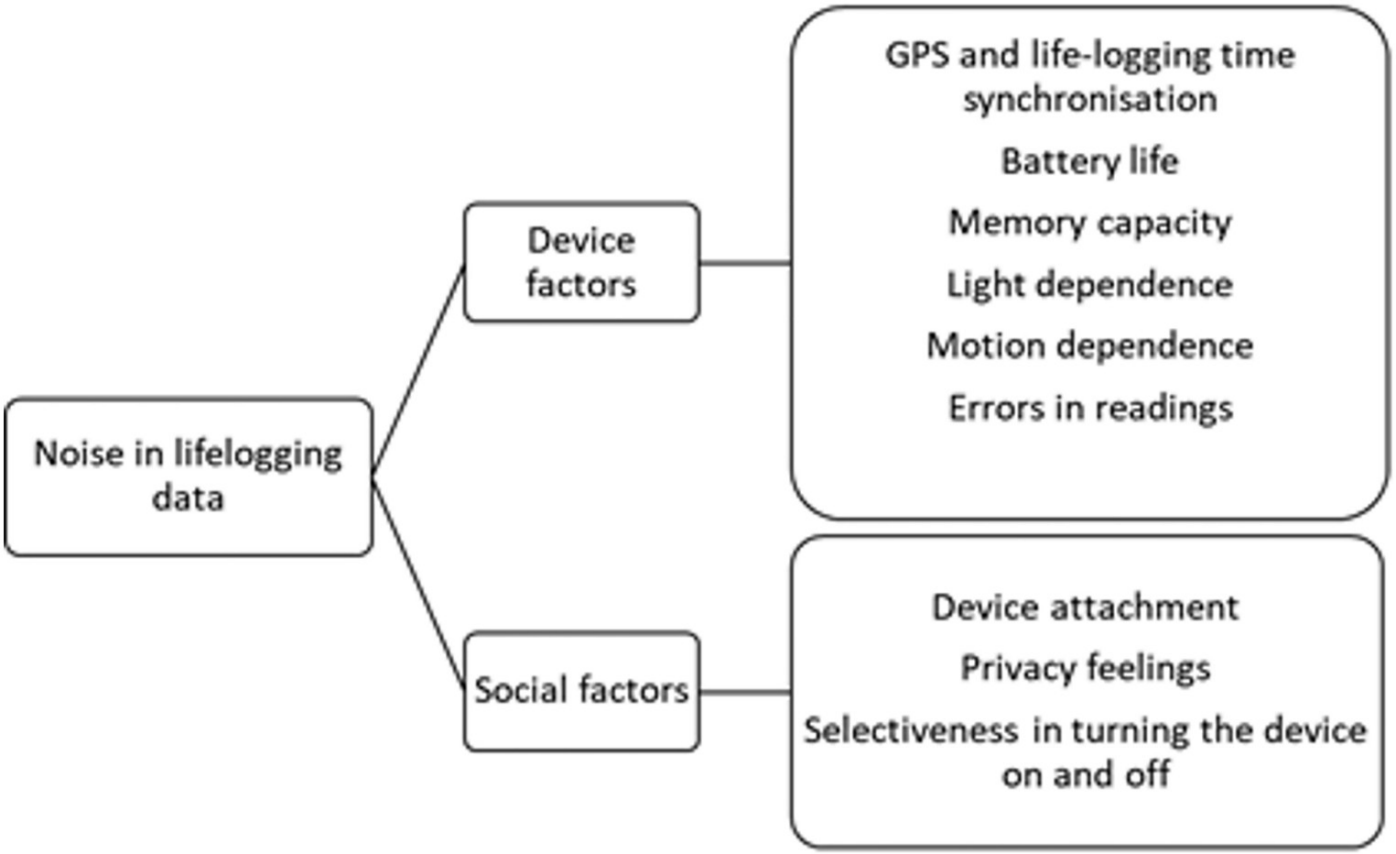
Noise sources in lifelogging data when linked to the GPS device.

The sensor data from a lifelogger are very sparse. The recording interval is on average 7 seconds which is much higher than a commonly used accelerometer in a phone or fitness tracker would have. Therefore, the classification of travel modes and activities is much harder Nevertheless, in our case, because we used additional sensors readings such as luminosity and temperature as well as the derived orientation we obtained satisfactory classification accuracies. Data from a wearable device such as a lifelogger can be very noisy. The noisiness can occur due to the factors relating to the device itself as well as social factors relating to the way people behave when carrying it. The devices occasionally stored sensor data as multiple entries with the same time stamp so, to reduce the problem and to still be able to use the images with the erroneous time stamps we substituted the identical values with values interpolated between first of the identical timestamps and the first after the identical timestamp. The device’s 10-hour battery life was another challenging factor meaning that it could not record a full day of data from a single charge. Lighting was another limitation due to the device’s camera being unable to capture photos when conditions were too dark therefore decreasing the number of images per person. Participants of the iMCD project were asked to switch the device off when entering certain locations such as: bathrooms, nurseries, schools and hospitals as well as to switch the device off and hide in case of adverse weather conditions such as heavy rain. We found that once they did they often tended to forget to turn the device back on which reduced the day’s data capture. Furthermore, they were advised to switch the device off in any other situation where they felt uncomfortable carrying it around, which again meant that the data collected was not always representative of a full and complete day of an individual but rather a sample of it. Fig 3 presents a list of possible noise sources in the data.

## Methodology: Development of traffic disturbance index

Driver inattention or agitation have long been recognized as the main contributing factors in traffic accidents [61,78]. The development of various intelligent driver assistance systems with embedded functionality for driver vigilance monitoring have therefore become an urgent and challenging task. In this paper we seek to identify local disturbance, inattention or agitation among drivers using multi-sensor data as well as analysis of the road network. By disturbance we mean the difference between the direction a car is heading and the driver’s body orientation.

### Linkage between GPS and lifelogging devices

To calculate disturbance we used an orientation calculation derived by the Autographer device’s manufacturer based on acceleration and magnetic field strength as well as the direction of travel on the road information from linked GPS data (more details below). The derived orientation was adjusted by a drift of 9 degrees calculated based on the experiment in which two mobile phones with an inbuilt gyroscope were used as well as the Autographer. In this experiment 100 measurements were taken for each of the three devices in different settings and orientations and based on the differences the average drift of 9 degrees was calculated. GPS data collected in the project were first cleaned and filtered to minimise the number of erroneous locations. Then the data were segmented into homogeneous parts using an algorithm developed by Authors [11]. Next, a two-step feedforward neural network with a general backpropagation algorithm was used for sub-trajectories classification; first to distinguish movement from non-movement segments and then to classify them into specific travel modes (driving, walking, bus and train). The GPS data classified as being in driving travel mode were map-matched to the OpenStreetMap road network using an existing Java-based library, Barefoot, which uses a Hidden Markov Model (HMM) method to deduce information about the object’s movement on the map. Each of the travel modes were assigned to GPS data points as an additional attribute. Both GPS device and Autographer were time synchronised to allow linkage by a timestamp. Even though the devices were synced, due to daylight saving and some other external factors, the linkage had to be manually verified to secure the highest accuracy. People were asked to carry the devices together for two consecutive day (most of them did it for one day only) but the devices did not appear to always be turned on at the same time, limiting the amount of linked data. To calculate accurate disturbance indices described in the next section, data that were classified as driving in both: GPS sample and lifelogging sample were linked together resulting in 26,652 records. These were data from a sample of 142 individuals who had a sufficient coverage (more than 30 minutes) of GPS and lifelogging data.

### Disturbance index

We define disturbance as the difference between the direction a car is heading and the body orientation of the driver. A car’s heading direction is derived following instructions in the steps described in Road direction (RD) and Individual Road Direction Identification (IRDI). The driver’s body orientation is the lifelogger’s orientation when attached to the driver in a forward-facing position, derived by the manufacturer using magnetometer and accelerometer readings.

**Fig 4:**
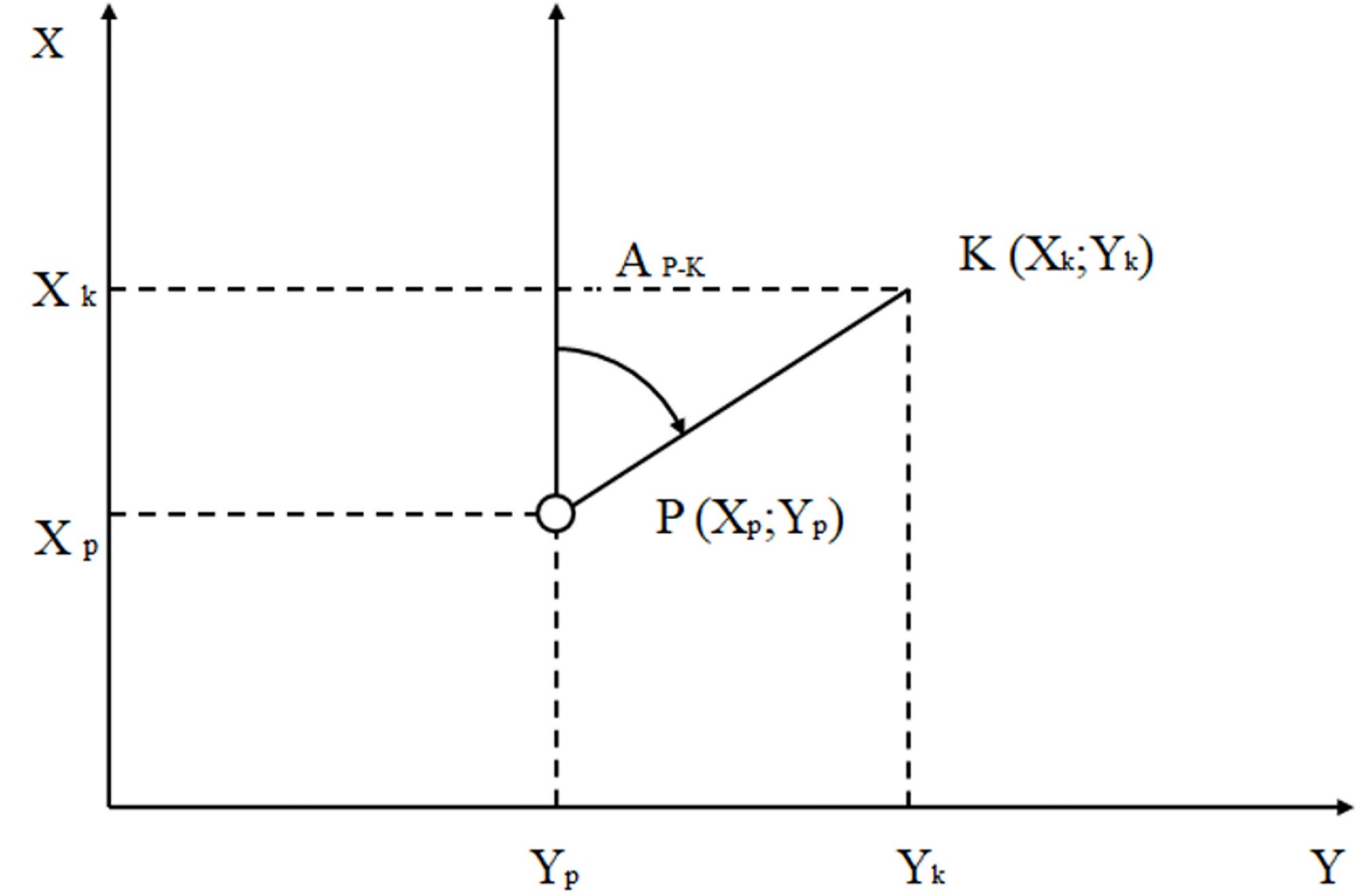
A schema for calculating an azimuth (A_P-K_) between points P_(Xp, Yp)_ and K_(Xk, Yk)._ Reference axes are swapped to the counter clockwise mathematical polar coordinates.

Road direction (RD)—The OSM road network was used. Each road link in the database is composed of a minimum of one straight road segment. To identify RD per each segment, these segments were separated and the direction (azimuth angle—A_PK_) of the road was calculated using the coordinates at the start and end of each section of the road (Fig 4).

An azimuth A_PK_ is a clockwise angular direction relative to the north (N) and point K. To calculate it, we first need to calculate differences in coordinates between points K and P:

$$\Delta X_{PK}\;=\;X_{K}-X_{P}$$ (5)

$$\Delta Y_{PK}\;=\;Y_{K}-Y_{P}$$ (6)

where X_i_ and Y_i_ are coordinates of points K and P. Next, depending on the sign of the above subtraction, a different set of rules applies:

$$if\;\Delta X_{PK}>0\;and\;\Delta Y_{PK}>0\;then\;A_{PK\;}\;=\;arctg\frac{\Delta Y_{PK}}{\Delta X_{PK}}$$ (7)

$$if\;\Delta X_{PK}>0\;and\;\Delta Y_{PK}>0\;then\;A_{PK\;}\;=\;arctg\frac{\Delta Y_{PK}}{\Delta X_{PK}}+360{}^{\circ}$$ (8)

$$if\;\Delta X_{PK}<0\;then\;A_{PK\;}\;=\;arctg\frac{\Delta Y_{PK}}{\Delta X_{PK}}+180$$ (9)

Exceptional cases:

$$\Delta X_{PK}\;=\;0\;and\;\{\begin{array}{c} \Delta Y_{PK}>0\;then\;A_{PK}\;=\;90{}^{\circ}\\ \Delta Y_{PK}<0\;then\;A_{PK}\;=\;270{}^{\circ} \end{array}\}$$ (10)

$$\Delta Y_{PK}\;=\;0\;and\;\{\begin{array}{c} \Delta X_{PK}>0\;then\;A_{PK}\;=\;0{}^{\circ}\\ \Delta X_{PK}<0\;then\;A_{PK}\;=\;180{}^{\circ} \end{array}\}$$ (11)

As it is a geometry-based calculation, it is impossible to know whether a calculated orientation of a road is real or has to be rotated by 180°. To minimise the risk of misclassification, a direction verification is suggested in the next step.

Individual Road Direction Identification—Heading (bearing—H_GPS_) from a map-matched set of GPS data allowed us to identify a direction towards which an individual is heading on a particular road (A_PK-GPS)_. A moving window was used to determine whether a sequence of consecutive GPS data points approached a start (P) or an end (K) of a road segment which identified the real direction of movement. If there was just one data point assigned to a particular road segment, then the next segment in a direction of either start or end was checked and verified and the headings for individual movements were assigned from a verified road direction. The original GPS-derived heading H_GPS_ was not used as it was calculated based only on the location of consecutive points, therefore prone to inaccuracy in the measurement of the actual direction of movement.

The difference between a road direction and heading (yaw) obtained from a lifelogging device carried by a participant in the project. Headings of a car (A_PK-GPS_) and individual (A_H_) are represented by an angular measure (0–360°). To calculate a difference (angle) between them we can use the following equations:

if A_PK-GPS_ ≥270° or A_H_ ≥270° then

$$d_{i,t}\;=\;abs(abs(A_{{PK_{GPS}}_{i,t}}-A_{H_{i,t}})-360{}^{\circ})$$ (12)

otherwise

$$d_{i,t}\;=\;abs(A_{{PK_{GPS}}_{i,t}}-A_{H_{i,t}})$$ (13)

where *d*_*i*,*t*_ is the disturbance attributed to participant *i* at time *t, $A_{{PK_{GPS}}_{i,t}}$* is a road azimuth verified by GPS heading and $A_{H_{i,t}}$ is the lifelogger’s orientation derived by the manufacturer using magnetometer and accelerometer readings. Although *d*_*i*,*t*_ can be greater than 180°, these cases were eliminated as erroneous as one cannot drive having their back turned to driving direction.

**Fig 5:**
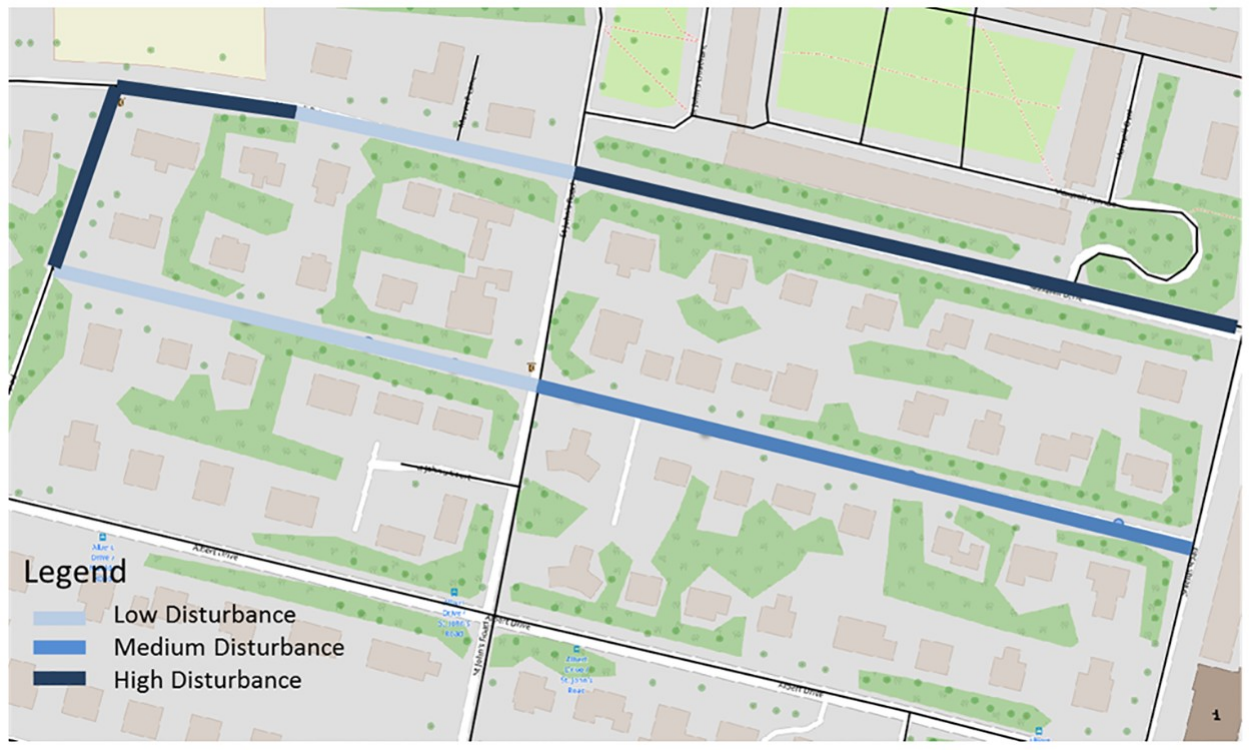
The visualisation of Index of disturbance calculates per each road link.

After calculating individual disturbances for each GPS point with the available sensor records we defined the *Index of Disturbance* per road link *d*_*tot*_ which can be written as:

$$d_{tot}\;=\;\frac{\sum_{1}^{n}d_{i,t}}{n}$$ (14)

where *n* is the number of GPS fixations with the calculated individual disturbance per road link in the network. The example of the index visualisation is presented in Fig 5.

The *Disturbance Index* was linked to the OSM road network from 2015. Table 6 presents the road classification used for this study.

**Table 6:** OSM road classification adjusted for the purposes of this paper. Source OSM Wiki.

Type of a road	Description
Motorway	A restricted access major divided highway, normally with 2 or more running lanes plus emergency hard shoulder.
Trunk roads	Strategic road connecting usually two cities, usually with two lines, no hard shoulder and possible traffic lights
Primary, Secondary, Tertiary—PST_Roads	The most important roads in a country’s system. A, B and C class roads.
Residential	Roads serving access to housing that do not connect settlements. Often lined with housing.
Unclassified	The least most important through roads in a country’s system

## Results—Indoor and outdoor activities—Results of model training and testing

### Indoor and outdoor movement for different socio-demographic groups

**Fig 6:**
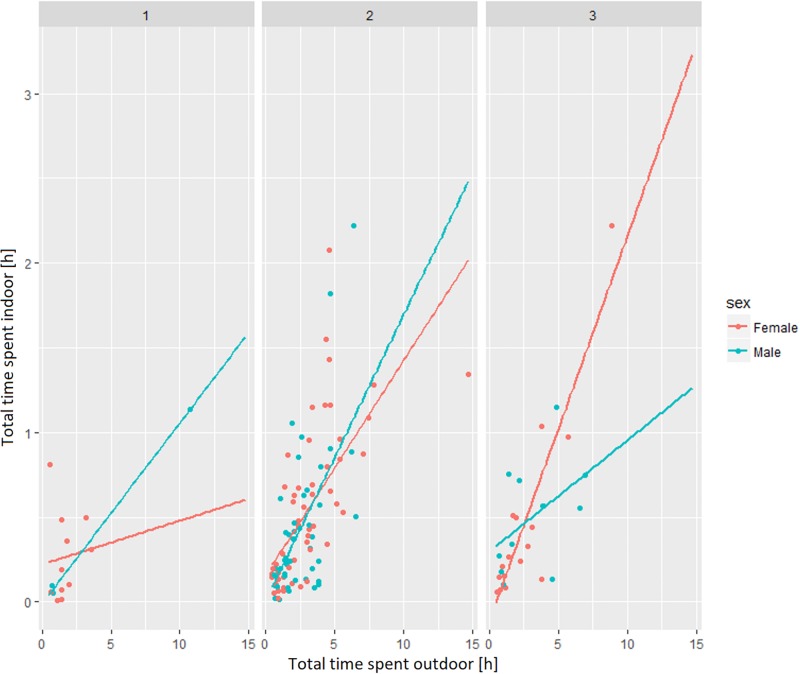
The relationship between time spent indoor and outdoor for different genders and age groups.

The results show a higher percentage of photos were taken in outdoor settings than indoor, which is incongruous with where the participants usually spent the most time. This suggests that they were more willing to use the wearable camera outdoors (driving is classified as an outdoor activity). This finding is similar to what Doherty et al. (58) found in their study. We found that younger men from the study (age group 1 and 2 –under 25 and between 25–65 respectively) tended to spend more time indoor than younger women, whereas for older people (65+) the reverse was true and women spent more time indoors then men (Fig 6).

**Fig 7:**
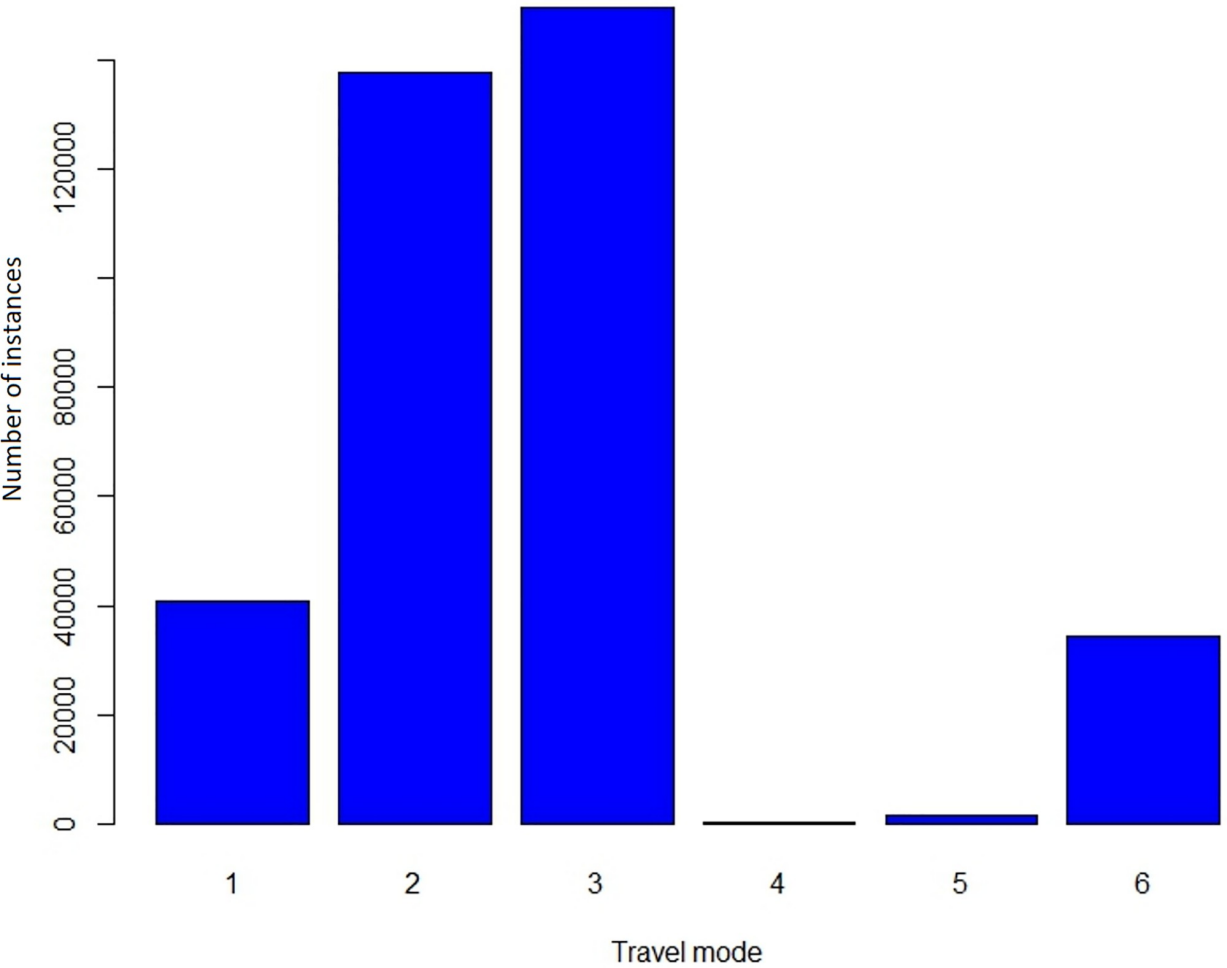
Distribution of the travel mode classes in the full data set of 139,254 lifelogging sensor readings.

The life-logging dataset was classified into six activity classes: 1—walking indoor, 2—walking outdoor, 3—driving outdoor, 4—sitting outdoor, 5—sitting indoor, 6—others (indoor class). There were not many (~4,000 records) sitting activities (either indoor or outdoor) classified from the dataset. The reason behind this is probably the variability in sitting positions and therefore most sitting activities were likely to have been classified as “others” (Fig 7). From the 142 individual participants analysed for this study, the average time per day spent on walking indoors is 22 minutes and 56 minutes on walking outdoors.

**Fig 8:**
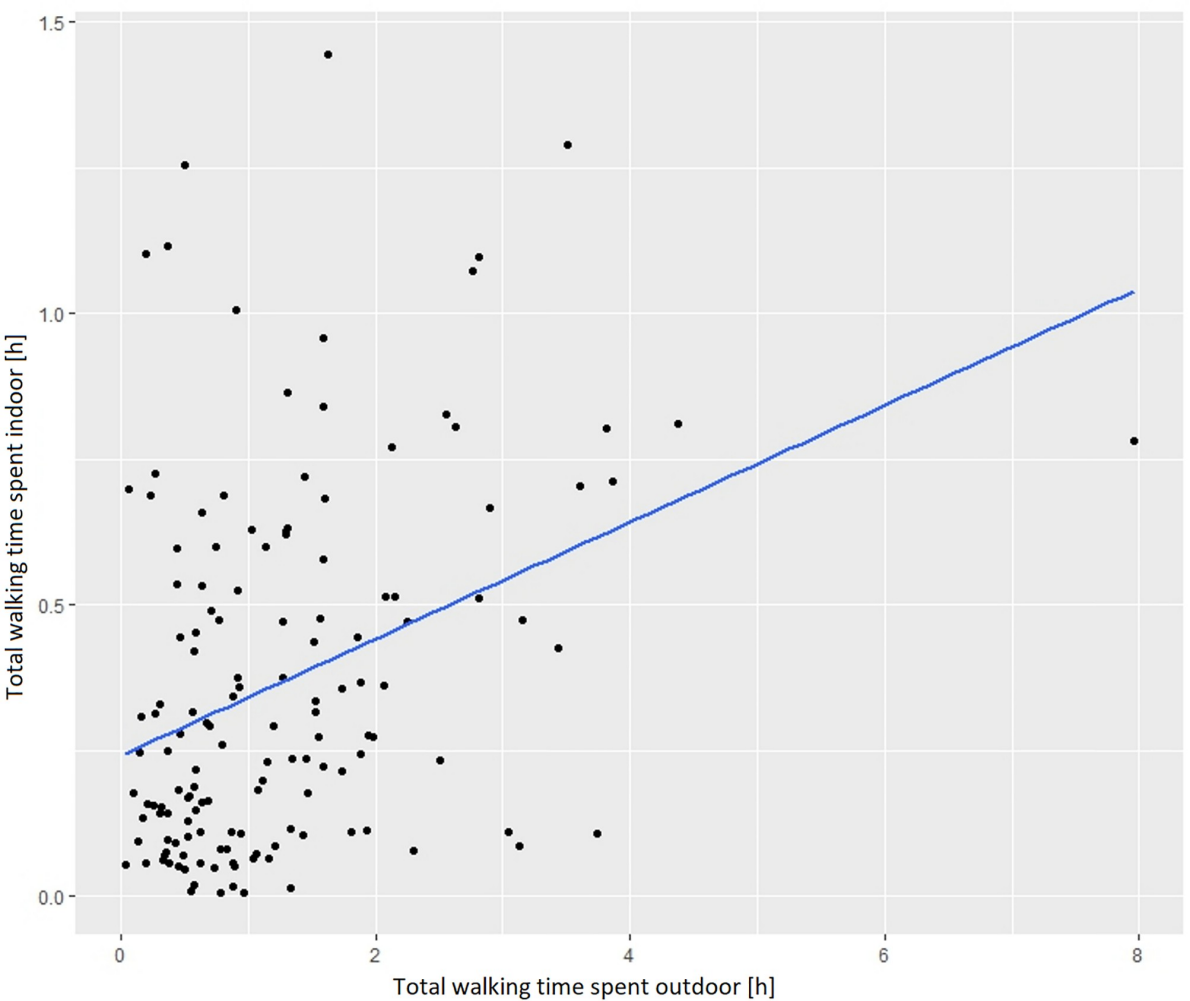
Relationship between indoor and outdoor walking activities.

**Fig 9:**
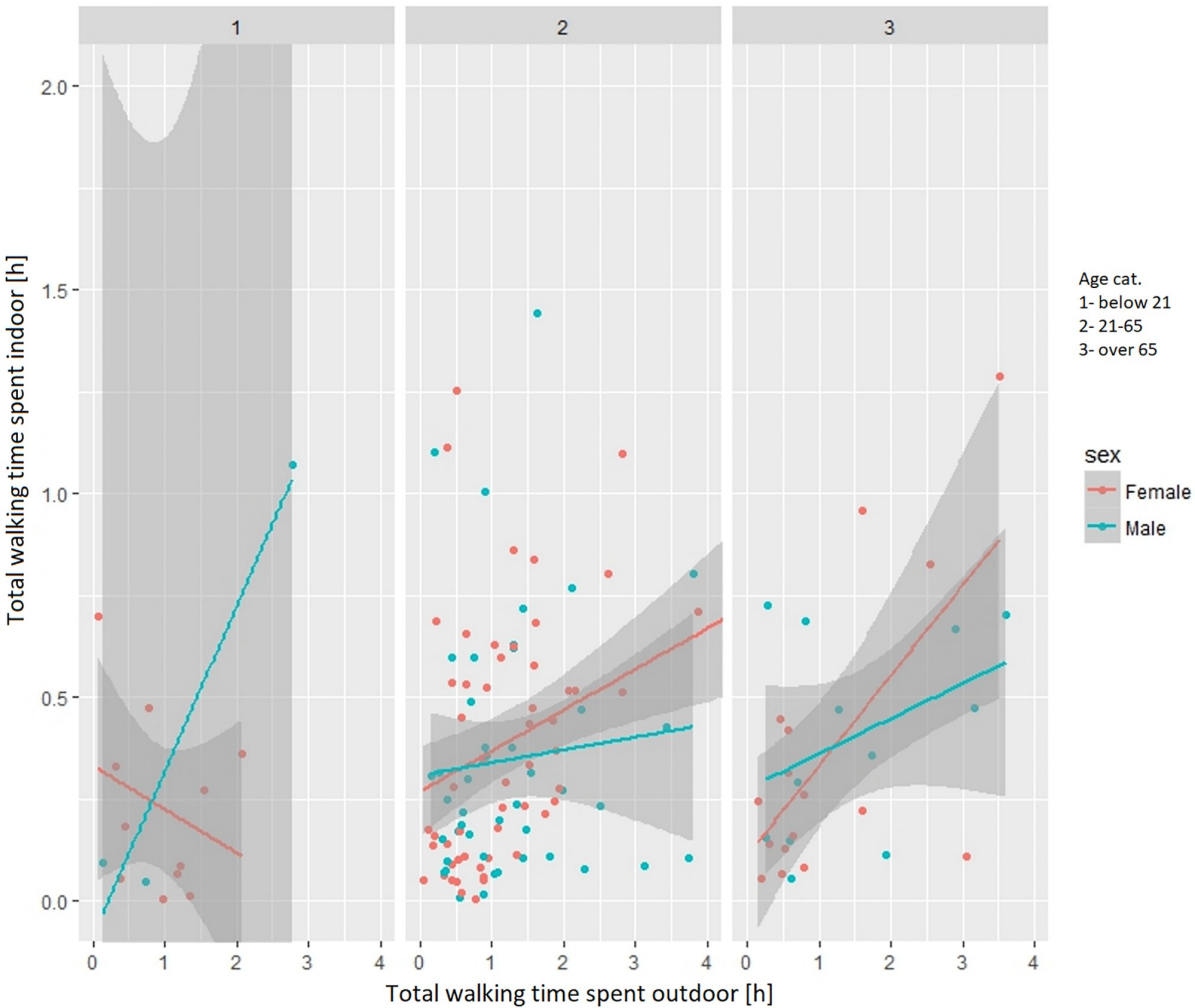
Relationship between indoor and outdoor walking for different genders and age groups.

Based on the collected sample of lifelogging data, the amount of time spent indoors increases with the amount of time spent outdoors (Fig 8), which means that the more people walk indoor, the more they walk outdoor as well. When looking at this relationship separately for men and women in different age groups various differences appear. For women under age 25 situation is opposite as they tend to walk less outdoor with an increase of indoor activities (Fig 9).

**Fig 10:**
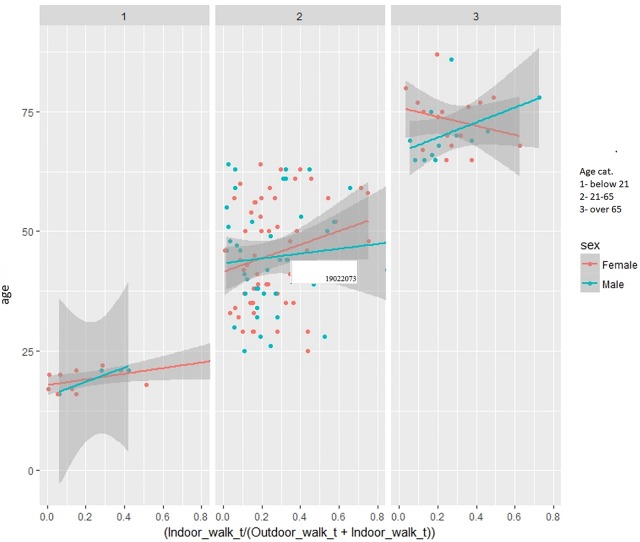
Percentage of indoor walking vs total walking per age group (1- under 25, 2–25–65 years old, 3–65+ years old).

As the total time of activities per day may not give an entirely accurate representation (some people have 12h of recording per day whereas others recorded just 4h), Fig 10 shows the relationship between age and indoor walking as a percentage of total walking activity. Men in all age groups tend to walk more as a percentage of total walking activity whereas for women this behaviour varies according to age. From this we can infer that the percentage of indoor walking increases with age where the only exception are older women.

## Results—Disturbance index

**Fig 11:**
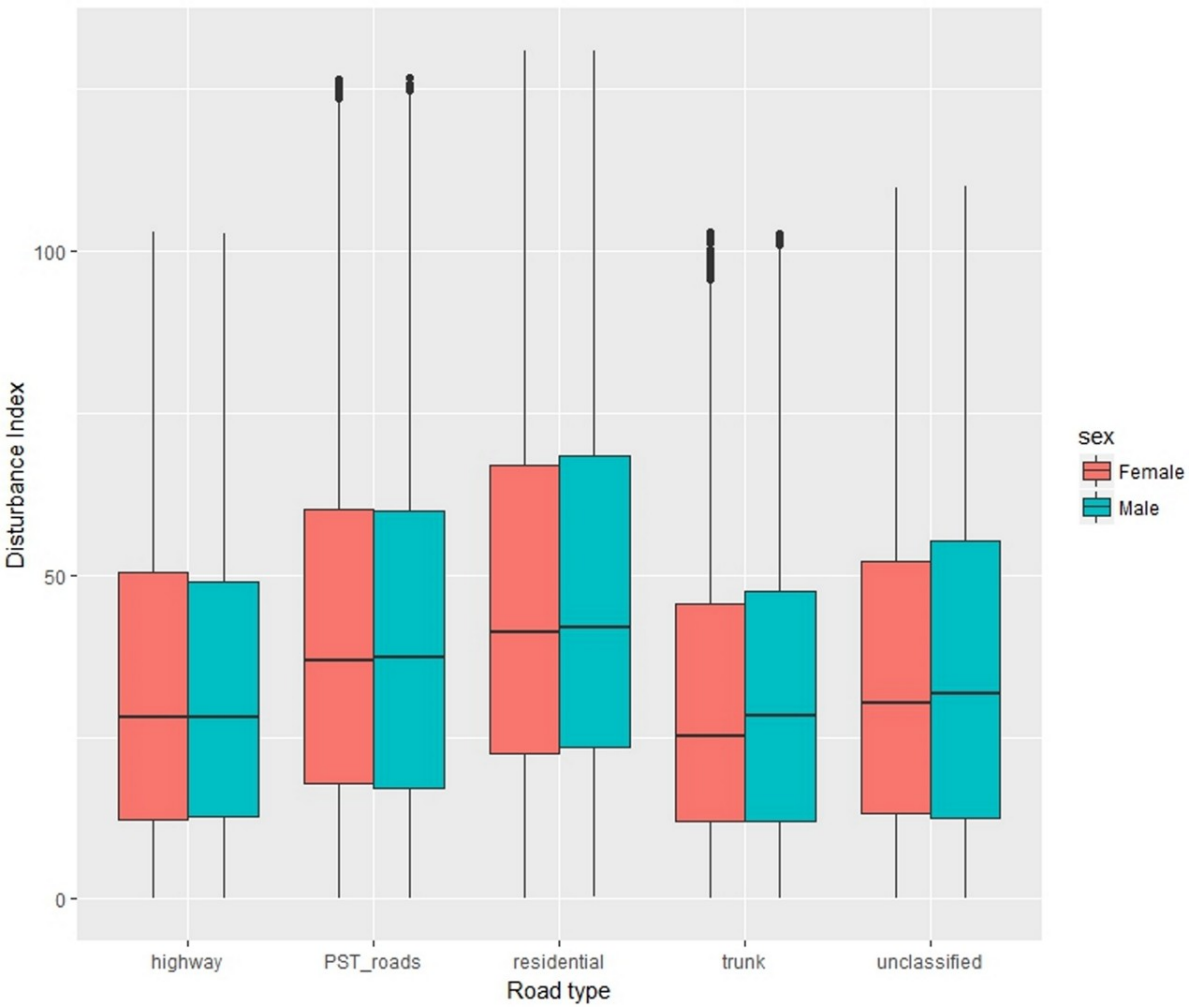
Individual disturbance for men and women on different roads.

*Individual Disturbances* were calculated only for the GPS data points where driving was detected as a travel mode, resulting in 26,652 movement locations across 5 road types (motorway, PST roads, residential, trunk and unclassified). The preliminary results show that there are differences between the levels of disturbance for men and women on different road types. It seems that on PST roads as well as residential roads the disturbance values seem to be higher both for men and women. Men have a slightly higher Disturbance Index than women in most of the road classes. It is also worth noting that for both men and women the levels of disturbance are lower on highways and trunk roads which are roads with higher speed limits and fewer interactions with potential external distractions and pedestrians (Fig 11).

**Fig 12:**
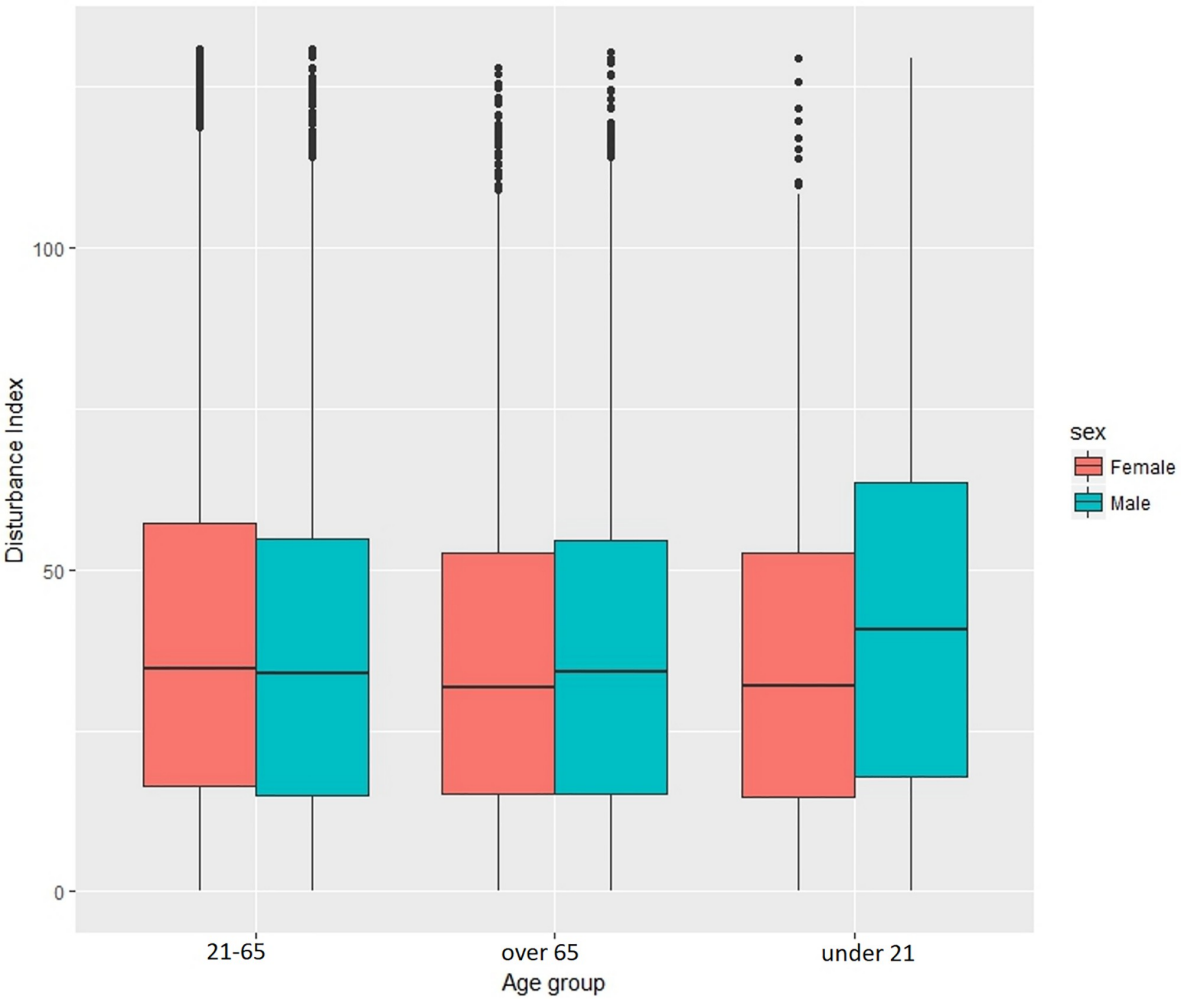
Individual disturbance for men and women in different age groups.

**Fig 13:**
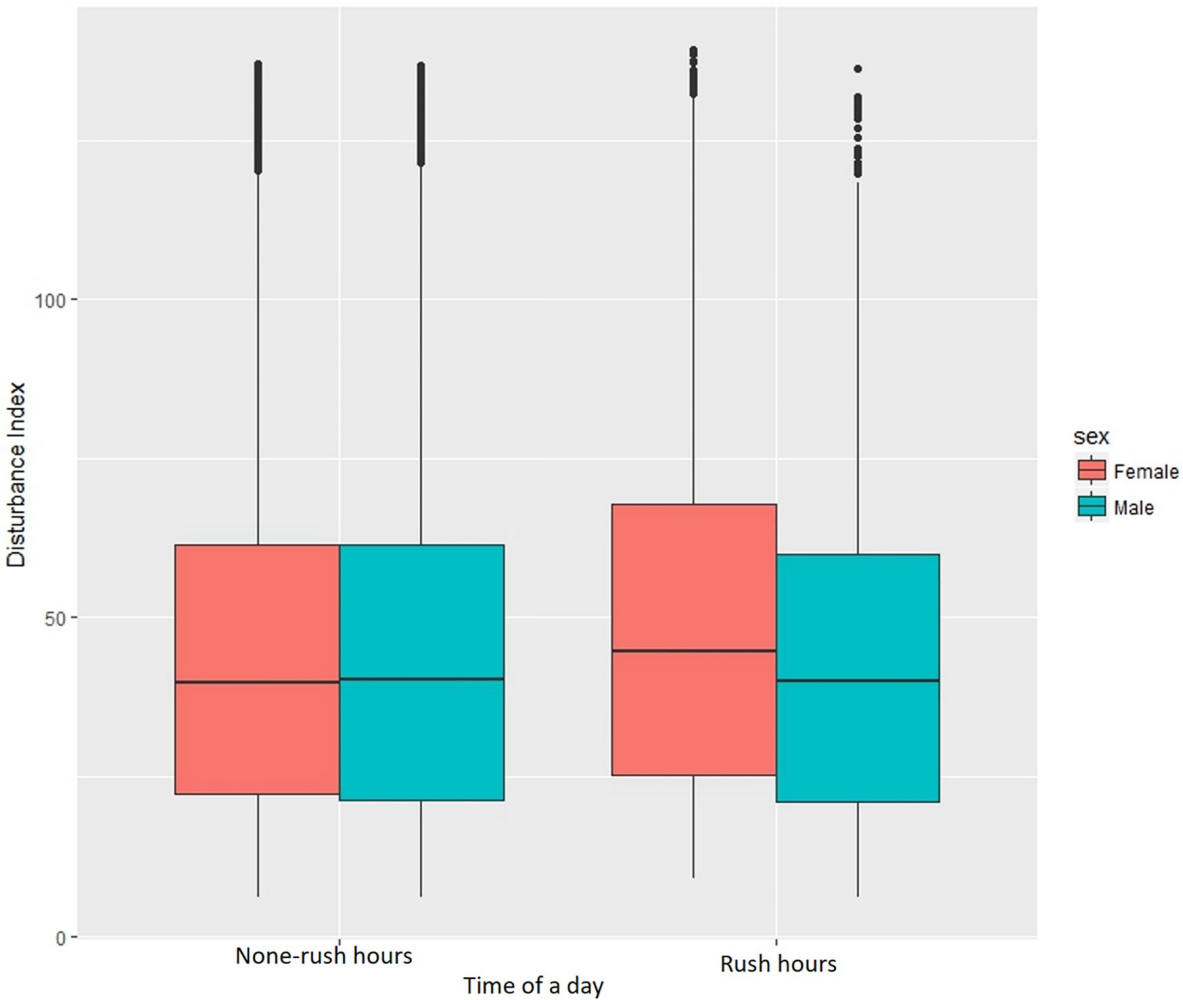
Individual disturbance in rush hour (7:30–9:00am and 4:00–6:00pm) and non-rush hours periods.

As traffic varies on the road during a day, there are also difference in the levels of disturbance between rush and non-rush hours periods. There does not seem to be a significant difference in driving behaviour for men and women in outside of rush hours periods but women have higher disturbance values when driving during the busier times (Figs 12 and 13)

**Fig 14:**
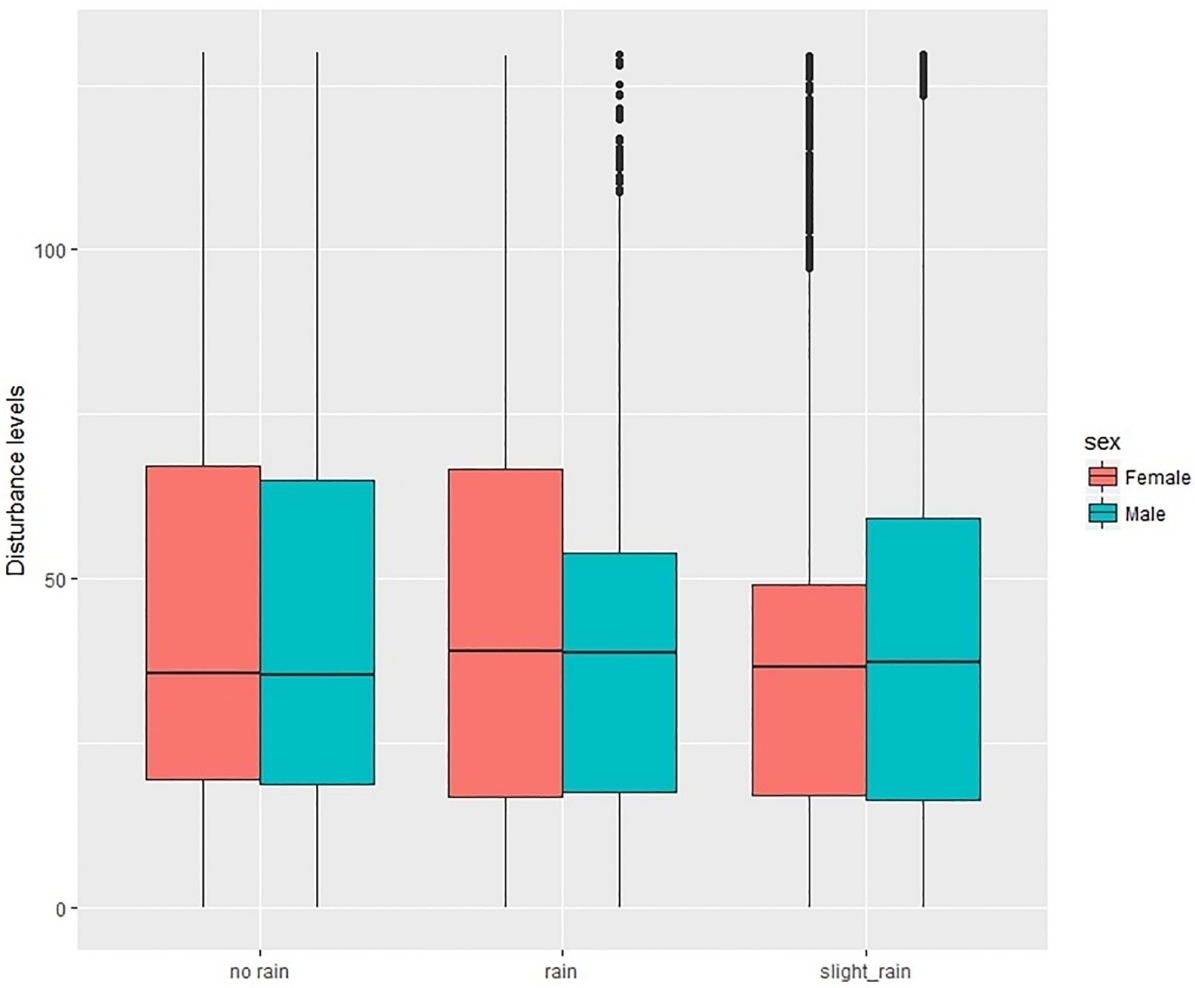
Individual disturbance for men and women in according to the weather conditions.

**Fig 15:**
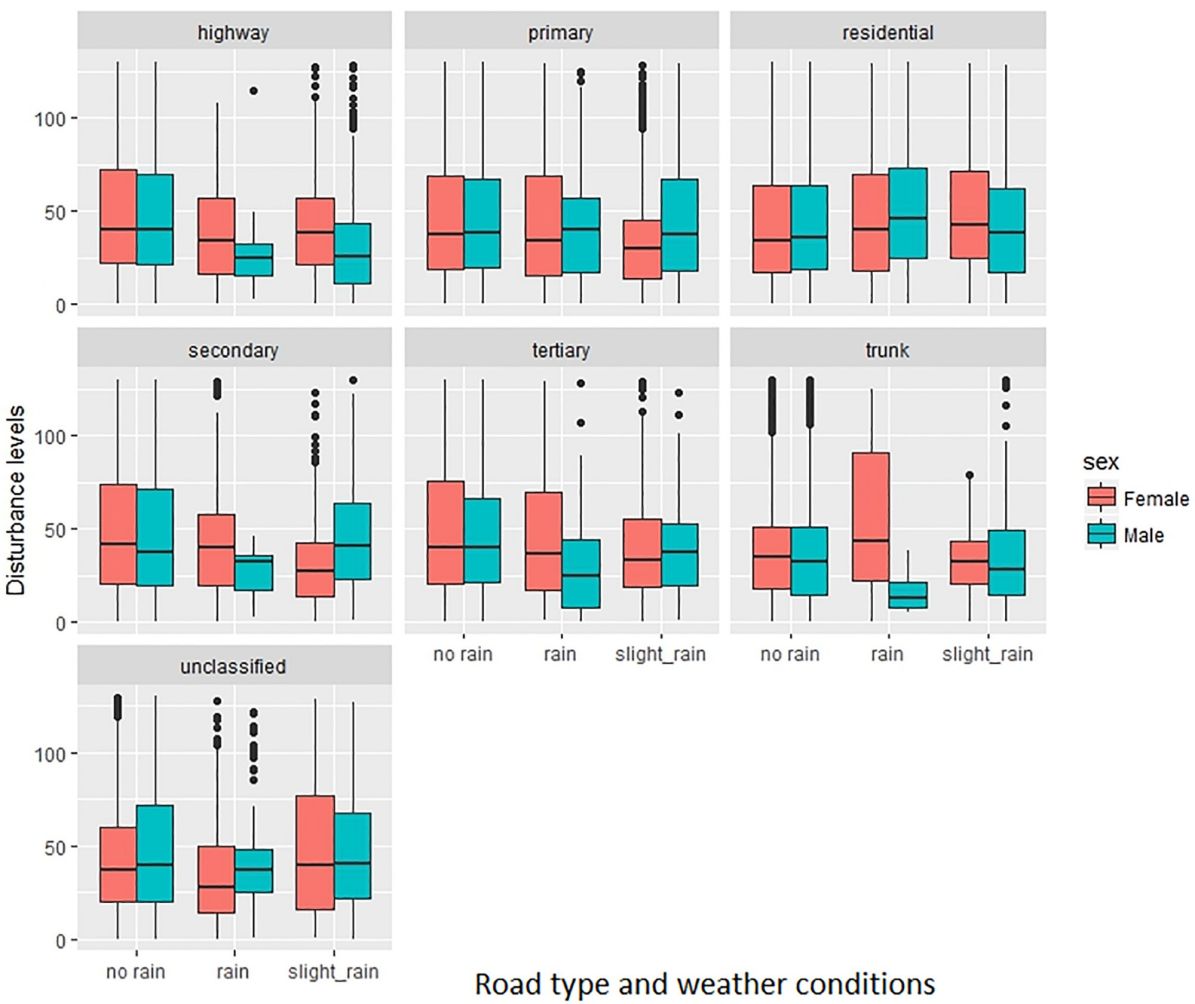
Individual disturbance for different road types according to different weather conditions.

As a last part of the experiment we linked weather data (wind and rainfall) from surrounding Glasgow meteorological stations (data obtained from Met Office). The weather data were collected in 30 minutes—1 hour intervals, so we interpolated the rain and wind values for each map-matched GPS data point. With the increased levels of rain categorised into three groups of heaviness–no rain, slight rain, and rain (more than 1mm/ sq m) we can clearly identify that the agitation/disturbance levels increase for both men and women with the amount of rain on residential and unclassified roads (Figs 14 and 15). The situation seems to be opposite on highways and primary to tertiary roads where the levels of agitation/disturbance are higher in dry conditions. This might be related to the fact that we drive much faster on highways when it is dry and we tend to overtake more than in rainy conditions. We did not find any relationship between the strength of wind and the level of disturbance index.

## Conclusions

In this paper we first solved the problem of indoor and outdoor movement detection from sensor readings associated with images captured by a lifelogger. Second we proposed a set of measures related with hazard on the road network derived from the combination of GPS movement data, road network data and the sensor readings from a wearable device. Third, we identified the relations between different socio-demographic groups and their indoor, outdoor walking activities as well as with the levels of disturbance while driving.

The indoor and outdoor activity detection performed in this paper showed that using sparse sensor data can result in high accuracy results. The results of our classification using multi-sensor readings are comparable to previous studies using only acceleration even though the readings interval is much higher. The relationship between the amount of indoor and outdoor activity shows that the more one walks outdoor the more one is active indoor as well. Furthermore, there are identified differences between mobility behaviour among men and women. The results presented in this study have certain limitations though, as the amount of time a survey participant was carrying the wearable device varied a lot what affects the representativeness of the results. Nevertheless, we plan to continue in more detail studying the relationship between outdoor and indoor activity levels with health-related characteristics of individuals from the iMCD survey.

The *Individual Disturbance* is a measure that allows to identify the level of disturbance or agitation at particular time and point on the road network. Our results show that there are differences in driving behaviour and the levels of disturbance according to different road types as well as different weather conditions. There are of course certain limitations to this study, as the measured disturbance does not take under consideration head movements but only the changes of direction of the chest. Therefore, it is possible that actual disturbance levels could be higher. Furthermore, due to the data availability limitations (we did not have data where people were not driving but were in the car) we are not able to distinguish whether an individual in the car is actually a driver or a passenger. This problem could be eliminated using object recognition image processing methods where we could identify a steering wheel form an image itself or with higher resolution accelerometer data where the small movements indicating changing gears or turning a steering wheel could be captured.

The suggested framework for identification of individual disturbance levels allows to reconstruct these steps for any similar data making this approach very scalable and potentially useful in road safety analysis. In future work, we will use the estimated individual disturbance levels and combine them with urban environment characteristics to identify potential reasons for disturbance or agitation on the roads.

## References

[1] Dodge M, Kitchin R. “Outlines of a world coming into existence”: Pervasive computing and the ethics of forgetting. Environ Plan B Plan Des [Internet]. SAGE PublicationsSage UK: London, England; 2007 6 22 [cited 2017 Sep 26];34(3):431–45. Available from: http://journals.sagepub.com/doi/10.1068/b32041t

[2] Gurrin C, Smeaton AF, Doherty AR. LifeLogging: Personal Big Data. Found Trends® Inf Retr. 2014;

[3] Fischer T, Riedl R. Lifelogging as a Viable Data Source for NeuroIS Researchers: A Review of Neurophysiological Data Types Collected in the Lifelogging Literature. In Springer, Cham; 2017 p. 165–74. http://link.springer.com/10.1007/978-3-319-41402-7_21

[4] Dabiri S, Heaslip K. Inferring transportation modes from GPS trajectories using a convolutional neural network. Transp Res Part C Emerg Technol [Internet]. Pergamon; 2018 1 1;86:360–71. Available from: https://www.sciencedirect.com/science/article/pii/S0968090X17303509

[5] Mäenpää H, Lobov A, Martinez Lastra JL. Travel mode estimation for multi-modal journey planner. Transp Res Part C Emerg Technol [Internet]. Pergamon; 2017 9 1;82:273–89. Available from: https://www.sciencedirect.com/science/article/pii/S0968090X17301808

[6] Abedi N, Bhaskar A, Chung E, Miska M. Assessment of antenna characteristic effects on pedestrian and cyclists travel-time estimation based on Bluetooth and WiFi MAC addresses. Transp Res Part C Emerg Technol [Internet]. Pergamon; 2015 11 1 [cited 2018 Feb 19];60:124–41. Available from: https://www.sciencedirect.com/science/article/pii/S0968090X15003113

[7] Shen L, Stopher PR. Review of GPS Travel Survey and GPS Data-Processing Methods. Transp Rev. 2014 4 4;34(3):316–34.

[8] Wolf J, Guensler R, Bachman W. Elimination of the travel diary: Experiment to derive trip purpose from global positioning system travel data. Transp Res Rec. Transportation Research Board of the National Academies; 2001 1 31;1768(1):125–34.

[9] Patterson DJ, Lin LA, Fox D, Kautz H. Inferring high-level behavior from low-level sensors. Ubicomp 2003 Ubiquitous Comput. 2003;2864:73–89.

[10] Gong H, Chen C, Bialostozky E, Lawson CT. A GPS/GIS method for travel mode detection in New York City. Comput Environ Urban Syst. 2012 3;36(2):131–9.

[11] Siła-Nowicka K, Vandrol J, Oshan T, Long JA, Demšar U, Fotheringham AS. Analysis of human mobility patterns from GPS trajectories and contextual information. Int J Geogr Inf Sci [Internet]. Taylor & Francis; 2016 5 3 [cited 2015 Nov 3];30(5):881–906. Available from: http://www.tandfonline.com/doi/full/10.1080/13658816.2015.1100731

[12] Xiao G, Juan Z, Zhang C. Detecting trip purposes from smartphone-based travel surveys with artificial neural networks and particle swarm optimization. Transp Res Part C Emerg Technol. 2016;71:447–63.

[13] Rundle AG, Sheehan DM, Quinn JW, Bartley K, Eisenhower D, Bader MMD, et al Using GPS Data to Study Neighborhood Walkability and Physical Activity. Am J Prev Med. 2016 11 7;50(3):65–72. 10.1016/j.amepre.2015.07.033 26558700

[14] Thakuriah P, Tilahun NY, Zellner M. Big Data and Urban Informatics: Innovations and Challenges to Urban Planning and Knowledge Discovery. In 2017 [cited 2018 May 17]. p. 11–45. http://link.springer.com/10.1007/978-3-319-40902-3_2

[15] Klepeis NE, Nelson WC, Ott WR, Robinson JP, Tsang AM, Switzer P, et al The National Human Activity Pattern Survey (NHAPS): a resource for assessing exposure to environmental pollutants. J Expo Sci Environ Epidemiol. 2001 7 26;11(3):231–52. 10.1038/sj.jea.7500165 11477521

[16] Sharifi MS, Stuart D, Christensen K, Chen A, Kim YS, Chen Y. Analysis of Walking Speeds Involving Individuals with Disabilities in Different Indoor Walking Environments. J Urban Plan Dev [Internet]. 2016 3 [cited 2018 Feb 27];142(1):4015010 Available from: http://ascelibrary.org/doi/10.1061/%28ASCE%29UP.1943-5444.0000288

[17] Prasertsubpakij D, Nitivattananon V. Evaluating accessibility to Bangkok Metro Systems using multi-dimensional criteria across user groups. IATSS Res [Internet]. Elsevier; 2012 7 1 [cited 2018 Feb 27];36(1):56–65. Available from: https://www.sciencedirect.com/science/article/pii/S0386111212000040

[18] Sharifi MS, Stuart D, Christensen K, Chen A. Time Headway Modeling and Capacity Analysis of Pedestrian Facilities Involving Individuals with Disabilities. Transp Res Rec J Transp Res Board [Internet]. 2016 1 [cited 2018 Feb 27];2553:41–51. Available from: http://trrjournalonline.trb.org/doi/10.3141/2553-05

[19] Gaire N. A Study on Human Evacuation Behavior Involving Individuals with Disabilities in a Building [Internet]. 2017 [cited 2018 Feb 27]. http://search.proquest.com/openview/c6a62c8f6016543fb47b64f660d23fe3/1?pq-origsite=gscholar&cbl=18750&diss=y

[20] Wu F, Zhu M, Wang Q, Zhao X, Chen W, Maciejewski R. Spatial–temporal visualization of city-wide crowd movement. J Vis [Internet]. Springer Berlin Heidelberg; 2017 5 9 [cited 2018 Feb 27];20(2):183–94. Available from: http://link.springer.com/10.1007/s12650-016-0368-4

[21] Antoniou C, Gikas V, Papathanasopoulou V, Mpimis T, Perakis H, Kyriazis C. A framework for risk reduction for indoor parking facilities under constraints using positioning technologies. Int J Disaster Risk Reduct [Internet]. Elsevier; 2017 9 18 [cited 2018 Feb 27]; https://www.sciencedirect.com/science/article/pii/S221242091730167X

[22] Kelly F, Nikopoulos CK. Facilitating independence in personal activities of daily living after a severe traumatic brain injury. Int J Ther Rehabil [Internet]. 2010 9 [cited 2018 Feb 27];17(9):474–82. Available from: http://www.magonlinelibrary.com/doi/10.12968/ijtr.2010.17.9.78037

[23] Spinney R, Smith L, Ucci M, Fisher A, Konstantatou M, Sawyer A, et al Indoor tracking to understand physical activity and sedentary behaviour: Exploratory study in UK office buildings. Shaman J, editor. PLoS One [Internet]. Public Library of Science; 2015 5 20;10(5):e0127688 Available from: http://dx.plos.org/10.1371/journal.pone.0127688 10.1371/journal.pone.0127688 25993515 PMC4684195

[24] Horberry T, Anderson J, Regan MA, Triggs TJ, Brown J. Driver distraction: The effects of concurrent in-vehicle tasks, road environment complexity and age on driving performance. Accid Anal Prev [Internet]. 2006 1 [cited 2018 Feb 27];38(1):185–91. Available from: http://linkinghub.elsevier.com/retrieve/pii/S0001457505001521 10.1016/j.aap.2005.09.007 16226211

[25] Young MS, Birrell SA, Stanton NA. Safe driving in a green world: A review of driver performance benchmarks and technologies to support “smart” driving. Appl Ergon [Internet]. Elsevier; 2011 5 1 [cited 2018 Mar 7];42(4):533–9. Available from: https://www.sciencedirect.com/science/article/pii/S0003687010001262 10.1016/j.apergo.2010.08.012 20863480

[26] Papantoniou P, Papadimitriou E, Yannis G. Review of driving performance parameters critical for distracted driving research. In: Transportation Research Procedia [Internet]. Elsevier; 2017 [cited 2018 Mar 7]. p. 1801–10. https://www.sciencedirect.com/science/article/pii/S2352146517304398

[27] Staton C, Vissoci J, Gong E, Toomey N, Wafula R, Abdelgadir J, et al Road Traffic Injury Prevention Initiatives: A Systematic Review and Metasummary of Effectiveness in Low and Middle Income Countries. Olivier J, editor. PLoS One [Internet]. Public Library of Science; 2016 1 6 [cited 2018 Feb 27];11(1):e0144971 Available from: http://dx.plos.org/10.1371/journal.pone.0144971 10.1371/journal.pone.0144971 26735918 PMC4703343

[28] Ledesma RD, Montes SA, Poó FM, López-Ramón MF. Measuring Individual Differences in Driver Inattention. Hum Factors J Hum Factors Ergon Soc [Internet]. 2015 3 18 [cited 2018 Feb 27];57(2):193–207. Available from: http://journals.sagepub.com/doi/10.1177/0018720814546530 10.1177/0018720814546530 25850151

[29] Steinberger F, Schroeter R, Watling CN. From road distraction to safe driving: Evaluating the effects of boredom and gamification on driving behaviour, physiological arousal, and subjective experience. Comput Human Behav [Internet]. Pergamon; 2017 10 1;75:714–26. Available from: http://www.sciencedirect.com/science/article/pii/S0747563217303904

[30] Zhang Z, Luo D, Rasim Y, Li Y, Meng G, Xu J, et al A Vehicle Active Safety Model: Vehicle Speed Control Based on Driver Vigilance Detection Using Wearable EEG and Sparse Representation. Sensors [Internet]. 2016 [cited 2018 Feb 27];16(2):242 Available from: http://www.mdpi.com/1424-8220/16/2/242/htm 10.3390/s16020242 26907278 PMC4801618

[31] Bi C, Huang J, Xing G, Jiang L, Liu X, Chen M. SafeWatch: A Wearable Hand Motion Tracking System for Improving Driving Safety. In: Proceedings of the Second International Conference on Internet-of-Things Design and Implementation [Internet]. 2017 [cited 2018 Feb 27]. p. 223–32. http://ieeexplore.ieee.org/abstract/document/7946880/

[32] Decker JS, Stannard SJ, McManus B, Wittig SMO, Sisiopiku VP, Stavrinos D. The Impact of Billboards on Driver Visual Behavior: A Systematic Literature Review. Traffic Inj Prev [Internet]. 2015 4 3 [cited 2018 Feb 27];16(3):234–9. Available from: https://www.tandfonline.com/doi/full/10.1080/15389588.2014.936407 25000270 10.1080/15389588.2014.936407 PMC4411179

[33] Dukic T, Ahlstrom C, Patten C, Kettwich C, Kircher K. Effects of Electronic Billboards on Driver Distraction. Traffic Inj Prev [Internet]. 2013 7 4 [cited 2018 Feb 27];14(5):469–76. Available from: http://www.tandfonline.com/doi/abs/10.1080/15389588.2012.731546 10.1080/15389588.2012.731546 23682577

[34] Loo BPY. The identification of Hazardous road locations: A comparison of the blacksite and hot zone methodologies in Hong Kong. Int J Sustain Transp [Internet]. 2009 [cited 2018 Mar 7];3(3):187–202. Available from: http://www.tandfonline.com/doi/abs/10.1080/15568310801915583

[35] Bíl M, Andrášik R, Janoška Z. Identification of hazardous road locations of traffic accidents by means of kernel density estimation and cluster significance evaluation. Accid Anal Prev [Internet]. 2013 [cited 2018 Mar 7];55:265–73. Available from: https://www.sciencedirect.com/science/article/pii/S0001457513000912 10.1016/j.aap.2013.03.003 23567216

[36] Elvik R. A survey of operational definitions of hazardous road locations in some European countries. Accid Anal Prev [Internet]. Pergamon; 2008 11 1 [cited 2018 Mar 7];40(6):1830–5. Available from: https://www.sciencedirect.com/science/article/pii/S0001457508001310 10.1016/j.aap.2008.08.001 19068283

[37] Bolanos M, Dimiccoli M, Radeva P. Toward Storytelling From Visual Lifelogging: An Overview. IEEE Trans Human-Machine Syst [Internet]. 2017 [cited 2017 Sep 26];1–14. http://ieeexplore.ieee.org/document/7723826/

[38] Jalal A, Kamal S. Real-time life logging via a depth silhouette-based human activity recognition system for smart home services. In: 11th IEEE International Conference on Advanced Video and Signal-Based Surveillance, AVSS 2014 [Internet]. IEEE; 2014 [cited 2017 Nov 6]. p. 74–80. http://ieeexplore.ieee.org/lpdocs/epic03/wrapper.htm?arnumber=6918647

[39] Wang P, Sun L, Yang S, Smeaton AF, Gurrin C. Characterizing everyday activities from visual lifelogs based on enhancing concept representation. Comput Vis Image Underst. Academic Press; 2016 7 1;148:181–92.

[40] Rew J, Hwang E, Choi YH, Rho S. Monitoring skin condition using life activities on the SNS user documents. Multimedia Tools and Applications. Springer US; 2017 4 22;1–21.

[41] Chambers T, Pearson AL, Stanley J, Smith M, Barr M, Ni Mhurchu C, et al Children’s exposure to alcohol marketing within supermarkets: An objective analysis using GPS technology and wearable cameras. Heal Place [Internet]. Pergamon; 2017 7 1;46:274–80. Available from: http://www.sciencedirect.com/science/article/pii/S1353829217304549 10.1016/j.healthplace.2017.06.003 28672147

[42] Dhand A, Dalton AE, Luke DA, Gage BF, Lee JM. Accuracy of Wearable Cameras to Track Social Interactions in Stroke Survivors. J Stroke Cerebrovasc Dis [Internet]. W.B. Saunders; 2016 12 1;25(12):2907–10. Available from: http://www.sciencedirect.com/science/article/pii/S1052305716302762 10.1016/j.jstrokecerebrovasdis.2016.08.004 27622865 PMC5154919

[43] Cornacchia M, Ozcan K, Zheng Y, Velipasalar S. A Survey on Activity Detection and Classification Using Wearable Sensors. IEEE Sens J [Internet]. 2017 1 15 [cited 2017 Jun 15];17(2):386–403. Available from: http://ieeexplore.ieee.org/document/7742959/

[44] Selke S. Lifelogging: Digital self-tracking and lifelogging—between disruptive technology and cultural transformation [Internet]. Lifelogging. Springer Fachmeiden Wiesbaden; 2016 [cited 2017 Sep 29]. 61–79 p. https://books.google.co.uk/books?hl=en&lr=&id=RxJkDAAAQBAJ&oi=fnd&pg=PP5&ots=lcVC4iswvJ&sig=owE0sKJh_OHvxr7g4eTkbLMz5Rg&redir_esc=y#v=onepage&q&f=false

[45] Bao L, Intille SS. Activity Recognition from user -annotated acceleration data In: PERVASIVE 2004. 2004 p. 1–17.

[46] Wang H, Calabrese F, Di Lorenzo G, Ratti C. Transportation mode inference from anonymized and aggregated mobile phone call detail records. IEEE Conf Intell Transp Syst Proceedings, ITSC [Internet]. 2010 [cited 2016 Feb 23];318–23. http://ieeexplore.ieee.org/xpls/abs_all.jsp?arnumber=5625188

[47] Awais M, Palmerini L, Chiari L. Physical activity classification using body-worn inertial sensors in a multi-sensor setup. In: 2016 IEEE 2nd International Forum on Research and Technologies for Society and Industry Leveraging a better tomorrow (RTSI) [Internet]. IEEE; 2016 [cited 2017 Jun 14]. p. 1–4. http://ieeexplore.ieee.org/document/7740565/

[48] van Hees VT, Gorzelniak L, Dean León EC, Eder M, Pias M, Taherian S, et al Separating Movement and Gravity Components in an Acceleration Signal and Implications for the Assessment of Human Daily Physical Activity. Müller M, editor. PLoS One [Internet]. 2013 4 23 [cited 2018 May 17];8(4):e61691 Available from: http://dx.plos.org/10.1371/journal.pone.0061691 10.1371/journal.pone.0061691 23626718 PMC3634007

[49] Bagalà F, Becker C, Cappello A, Chiari L, Aminian K, Hausdorff JM, et al Evaluation of accelerometer-based fall detection algorithms on real-world falls. Bayer A, editor. PLoS One [Internet]. 2012 5 16 [cited 2018 May 17];7(5):e37062 Available from: http://dx.plos.org/10.1371/journal.pone.0037062 10.1371/journal.pone.0037062 22615890 PMC3353905

[50] Shafique MA, Hato E. Use of acceleration data for transportation mode prediction. Transportation (Amst) [Internet]. Springer US; 2015 1 1 [cited 2016 Oct 19];42(1):163–88. Available from: http://link.springer.com/10.1007/s11116-014-9541-6

[51] Lim D, Park C, Kim NH, Kim S-H, Yu YS. Fall-Detection Algorithm Using 3-Axis Acceleration: Combination with Simple Threshold and Hidden Markov Model. J Appl Math [Internet]. Hindawi Publishing Corporation; 2014 [cited 2017 Mar 29];2014:1–8. http://www.hindawi.com/journals/jam/2014/896030/

[52] Asimakopoulos S, Asimakopoulos G, Spillers F. Motivation and User Engagement in Fitness Tracking: Heuristics for Mobile Healthcare Wearables. Informatics [Internet]. Multidisciplinary Digital Publishing Institute; 2017 1 22 [cited 2017 Oct 2];4(1):5 http://www.mdpi.com/2227-9709/4/1/5

[53] Patel S, Park H, Bonato P, Chan L, Rodgers M. A review of wearable sensors and systems with application in rehabilitation. J Neuroeng Rehabil [Internet]. BioMed Central; 2012 4 20 [cited 2017 Oct 2];9(1):21 Available from: http://jneuroengrehab.biomedcentral.com/articles/10.1186/1743-0003-9-21 22520559 10.1186/1743-0003-9-21 PMC3354997

[54] Piwek L, Ellis DA, Andrews S, Joinson A. The Rise of Consumer Health Wearables: Promises and Barriers. PLOS Med [Internet]. Public Library of Science; 2016 2 2 [cited 2017 Oct 2];13(2):e1001953 Available from: http://dx.plos.org/10.1371/journal.pmed.1001953 10.1371/journal.pmed.1001953 26836780 PMC4737495

[55] Wright R, Keith L. Wearable Technology: If the Tech Fits, Wear It. J Electron Resour Med Libr [Internet]. Taylor & Francis Group; 2014 10 2 [cited 2017 Oct 2];11(4):204–16. Available from: http://www.tandfonline.com/doi/abs/10.1080/15424065.2014.969051

[56] El Asnaoui K, Hamid A, Brahim A, Mohammed O. A survey of activity recognition in egocentric lifelogging datasets. In: 2017 International Conference on Wireless Technologies, Embedded and Intelligent Systems, WITS 2017 [Internet]. IEEE; 2017 [cited 2017 Jul 18]. p. 1–8. http://ieeexplore.ieee.org/document/7934659/

[57] Wang Z, Yang Z, Dong T. A review of wearable technologies for elderly care that can accurately track indoor position, recognize physical activities and monitor vital signs in real time [Internet]. Sensors (Switzerland). 2017 [cited 2017 Sep 26]. p. 341 http://www.ncbi.nlm.nih.gov/pubmed/28208620 10.3390/s17020341 PMC5336038 28208620

[58] Doherty AR, Kelly P, Kerr J, Marshall S, Oliver M, Badland H, et al Using wearable cameras to categorise type and context of accelerometer-identified episodes of physical activity. Int J Behav Nutr Phys Act [Internet]. BioMed Central; 2013 2 13 [cited 2018 Mar 7];10(1):22 Available from: http://ijbnpa.biomedcentral.com/articles/10.1186/1479-5868-10-22 10.1186/1479-5868-10-22 PMC3615956 23406270

[59] Scott M, Follmer T, Jonathan CC. SYSTEM AND METHOD FOR MONITORING AND IMPROVING DRIVER BEHAVIOR. Google Patents [Internet]. 2008 [cited 2018 Mar 8];1(19). Available from: https://patents.google.com/patent/US9129460B2/en

[60] Kulkarni AS, Shinde SB. Monitoring Driver Distraction in Real Time using Computer Vision System. ieeexplore.ieee.org [Internet]. 2017 [cited 2018 Mar 8];(6):121–8. http://ieeexplore.ieee.org/abstract/document/8191851/

[61] Lu SN, Tseng HW, Lee YH, Jan YG, Lee WC. Intelligent safety warning and alert system for car driving. Tamkang J Sci Eng. 2010;13(4):395–404.

[62] Koesdwiady A, Soua R, Karray F, Kamel MS. Recent Trends in Driver Safety Monitoring Systems: State of the Art and Challenges. IEEE Trans Veh Technol [Internet]. 2017 6 [cited 2018 Mar 8];66(6):4550–63. Available from: http://ieeexplore.ieee.org/document/7752938/

[63] Jung S-J, Shin H-S, Chung W-Y. Driver fatigue and drowsiness monitoring system with embedded electrocardiogram sensor on steering wheel. IET Intell Transp Syst [Internet]. 2014 [cited 2018 Mar 8];8(1):43–50. Available from: http://ieeexplore.ieee.org/abstract/document/6720253/

[64] Dong Y, Hu Z, Uchimura K, Murayama N. Driver inattention monitoring system for intelligent vehicles: A review. In: IEEE Transactions on Intelligent Transportation Systems [Internet]. 2011 [cited 2018 Apr 16]. p. 596–614. http://ieeexplore.ieee.org/document/5665773/

[65] Oviedo-Trespalacios O, Haque MM, King M, Washington S. Effects of road infrastructure and traffic complexity in speed adaptation behaviour of distracted drivers. Accid Anal Prev [Internet]. Pergamon; 2017 4 1 [cited 2018 Jan 9];101:67–77. Available from: https://www.sciencedirect.com/science/article/pii/S0001457517300453 10.1016/j.aap.2017.01.018 28189943

[66] Martín de Diego I, Siordia S. O, Crespo R, Conde C, Cabello E. Transp Res Part C Emerg Technol [Internet]. Pergamon; 2013 1 1 [cited 2018 Jan 15];26:380–95. Available from: https://www.sciencedirect.com/science/article/pii/S0968090X12001283

[67] Griffin R, Huisingh C, McGwin G. Prevalence of and Factors Associated with Distraction Among Public Transit Bus Drivers. Traffic Inj Prev [Internet]. 2014 10 3;15(7):720–5. Available from: http://www.ncbi.nlm.nih.gov/pubmed/24433192 10.1080/15389588.2013.867482 24433192 PMC4391701

[68] Rogers M, Zhang Y, Kaber D, Liang Y, Gangakhedkar S. The Effects of Visual and Cognitive Distraction on Driver Situation Awareness. In Springer, Berlin, Heidelberg; 2011 p. 186–95. http://link.springer.com/10.1007/978-3-642-21741-8_21

[69] Thakuriah PV, Geers DG. Transportation and Information: Trends in Technology and Policy [Internet]. Springer. 2013 [cited 2018 May 17]. 127 p. https://link.springer.com/content/pdf/10.1007/978-1-4614-7129-5.pdf

[70] Thakuriah P, Sila-Nowicka K, Paule J. Sensing Spatiotemporal Patterns in Urban Areas: Analytics and Visualizations using the Integrated Multimedia City Data Platform. Built Environ. 2016;42(3):415–29.

[71] NHS website [Internet]. https://www.nhs.uk/live-well/healthy-weight/height-weight-chart/

[72] Transystem Inc. Transmit GPS manual [Internet]. 2011 [cited 2018 Sep 25]. http://www.transystem.com.tw/www/product.php?b=G&m=pe&cid=4&sid=&id=96

[73] Huang Z. Extensions to the k-Means Algorithm for Clustering Large Data Sets with Categorical Values. Data Min Knowl Discov [Internet]. Kluwer Academic Publishers; 1998 [cited 2018 Mar 14];2(3):283–304. Available from: http://link.springer.com/10.1023/A:1009769707641

[74] Attal F, Mohammed S, Dedabrishvili M, Chamroukhi F, Oukhellou L, Amirat Y. Physical Human Activity Recognition Using Wearable Sensors. Sensors [Internet]. 2015;15(12):31314–38. Available from: http://www.mdpi.com/1424-8220/15/12/29858 10.3390/s151229858 26690450 PMC4721778

[75] Breiman L. Random forests. Mach Learn [Internet]. 2001 [cited 2018 Mar 16];45(1):5–32. Available from: https://link.springer.com/article/10.1023/A:1010933404324

[76] Fawagreh K, Gaber MM, Elyan E. Random forests: from early developments to recent advancements. Syst Sci Control Eng [Internet]. Taylor & Francis; 2014 12 6 [cited 2018 Mar 16];2(1):602–9. Available from: http://www.tandfonline.com/doi/abs/10.1080/21642583.2014.956265

[77] Arif M, Kattan A, Moore S, Howard D, Lyons D. Physical Activities Monitoring Using Wearable Acceleration Sensors Attached to the Body. Lucia A, editor. PLoS One[Internet]. Springer; 2015 7 23 [cited 2017 Mar 29];10(7):e0130851 Available from: http://dx.plos.org/10.1371/journal.pone.0130851 10.1371/journal.pone.0130851 26203909 PMC4512690

[78] Galéra C, Gil-Jardinet C, Née M, Salmi R, Orriols L, Contrand B, et al 764 The impact of working memory and selective attention on road safety. Inj Prev [Internet]. BMJ Publishing Group Ltd; 2016 9 1;22(Suppl 2):A273.3–A274. Available from: http://injuryprevention.bmj.com/lookup/doi/10.1136/injuryprev-2016-042156.764

